# Siglec-9, a Putative Immune Checkpoint Marker for Cancer Progression Across Multiple Cancer Types

**DOI:** 10.3389/fmolb.2022.743515

**Published:** 2022-03-17

**Authors:** Yuliang Wu, Wei Huang, Yutong Xie, Chunyan Wang, Ning Luo, Yingying Chen, Liefu Wang, Zhongping Cheng, Zhengliang Gao, Shupeng Liu

**Affiliations:** ^1^ Department of Obstetrics and Gynecology, Shanghai Tenth People’s Hospital, Tongji University, Shanghai, China; ^2^ Yangzhi Rehabilitation Hospital (Shanghai Sunshine Rehabilitation Center), Tongji University School of Medicine, Shanghai, China; ^3^ Gynecologic Minimally Invasive Surgery Research Center, Tongji University School of Medicine, Shanghai, China; ^4^ Xinyang Vocational and Technical College, Xinyang, China; ^5^ Institute of Geriatrics (Shanghai University), Affiliated Nantong Hospital of Shanghai University (The Sixth People’s Hospital of Nantong), School of Medicine, Shanghai University, Nantong, China; ^6^ Department of Obstetrics and Gynecology, Putuo District People’s Hospital of Shanghai City, Shanghai, China

**Keywords:** Siglec-9, the Cancer Genome Atlas, immune infiltration, pan-cancer, LGG, GSEA

## Abstract

Siglec-9, a cell surface transmembrane receptor mainly expressed on B cells, CD56^+^ NK cells, and CD4^+^ and CD8^+^ T cells, is strongly related to the tumor immune microenvironment. However, the expression pattern of Siglec-9 and its prognostic potential have not been investigated in a pan-cancer perspective. This study aimed to explore the association of Siglec-9 with prognosis, tumor stage, molecular subtype, and the immune microenvironment in pan-cancer. The mRNA expression of Siglec-9 was obtained from The Cancer Genome Atlas (TCGA), the Broad Institute Cancer Cell Line Encyclopedia (CCLE), and Genotype-Tissue Expression (GTEx). The relationship between Siglec-9 mRNA expression and prognosis was evaluated by the Kaplan–Meier analysis. The correlation between Siglec-9 and tumor-infiltrating immune cells, immune subtype, and molecular subtype was evaluated on Tumor Immune Estimation Resource (TIMER) and Integrated Repository Portal for Tumor-Immune System Interactions (TISIDB). The correlation between Siglec-9 expression and immune checkpoint, mismatch repair (MMR), DNA methyltransferase (DNMT), tumor mutation burden (TMB), and microsatellite instability (MSI) was also analyzed. It showed that Siglec-9 expression was significantly altered in most TCGA tumors. Siglec-9 expression was associated with the prognosis of patients with adrenocortical carcinoma (ACC), lung adenocarcinoma (LUSC), thymoma (THYM), colon adenocarcinoma (COAD), glioblastoma multiforme (GBM), prostate adenocarcinoma (PRAD), esophageal carcinoma (ESCA), and brain lower-grade glioma (LGG). Particularly, increased Siglec-9 expression was strongly correlated with poor prognosis in LGG. Correlation between Siglec-9 expression and tumor stage was also observed in various cancers. In addition, Siglec-9 was positively associated with infiltration of immune cells including neutrophils, dendritic cells (DCs), macrophage, and CD4^+^ and CD8^+^ T cells. Moreover, a significant correlation between Siglec-9 and MSI, TMB, MMR, DNMT, immune checkpoint, immune subtype, molecular subtype, and immunomodulators was observed in multiple cancers. Specifically, poor prognostic value and strong correlation to immune cell infiltration were verified with the LGG dataset from the Chinese Glioma Genome Atlas (CGGA). These findings indicated that Siglec-9 can be a novel biomarker and a potential target for cancer immunotherapy.

## Introduction

Tumors remain to be a medical problem seriously endangering human health (Bray et al., 2020). Therapies including surgery, chemotherapy, and radiotherapy improved the prognosis of patients, but millions of deaths were induced by tumor relapse and metastasis. Immunodeficiency and immunosuppression are among the main causes of tumorigenesis and recurrence (Mortezaee, 2020). Immunotherapy including immune checkpoint therapy and chimeric antigen receptor T (CAR-T) cell therapy has improved prognosis in tumors such as non-small-cell lung cancer (NLSLC), OV, and B-cell lymphoma (Neelapu et al., 2017; Galluzzi et al., 2018). However, immune therapy remains a poor therapeutic effect in solid tumors, with the underlying mechanism remaining unclear.

Immune checkpoints are involved in tumor immunosuppression and are supposed to be ideal modulation targets for tumor immunotherapy. The sialic acid-binding immunoglobulin-like lectins (Siglecs), novel kinds of immune checkpoints involved in tumor immunosuppression, may be new targets, or biomarkers or prognostic factors of immunotherapy (Liu et al., 2021). Siglecs family, belonging to the immunoglobulin superfamily, is a type I transmembrane protein of I-lectin that consists of a V-set immunoglobulin blind domain and a series of C2-set Ig-like domain in the extracellular zone^7^. Siglecs are mainly expressed on immune cells and manipulate immune function *via* recognizing sialylated glycans (Zheng et al., 2020). Siglecs can arouse inhibitory signals to immune cells in either an in-cis or an in-trans manner (Fraschilla and Pillai, 2017). Among Siglecs, Siglec-9, one of the Siglecs with ITIM domains, was found to be widely involved in tumor progression and tumor immunosuppression (Fraschilla and Pillai, 2017). It was reported to deliver activation signals to cancer cells and activate inhibitory signals in immune cells (Sabit et al., 2013). Siglec-9 promoted the growth of adenocarcinomas or hematological tumor cells *via* recruiting β-catenin to the C-terminal domain of Siglec-9 by interacting with MUC1 on tumor cells (Tanida et al., 2013). It also reported that Siglec-9 promoted cancer cell growth by interacting with MUC16 on epithelial ovarian cancer cells (Belisle et al., 2010). In addition to the direct effect on tumor cells, activation of Siglec-9 on NK cells, B cells, T cells, and monocytes was reported to restrain cytolytic capacity and helped tumor cells evade immune surveillance (Belisle et al., 2010). Combining with the finding that Siglec-9 was wildly expressed on immune cells including neutrophils, NK cells, B cells, macrophages, and monocytes, it suggests that Siglec-9 might be a potential target for cancer immunotherapy and a potential prognostic marker of cancer patients. However, little is known about the expression pattern and the prognostic value of Siglec-9 in cancers.

In the present study, datasets from The Cancer Genome Atlas (TCGA) and Genotype-Tissue Expression (GTEx) were obtained and used to assess the expression pattern of Siglec-9 and its correlation with prognosis and tumor immune microenvironment. It showed that the expression of Siglec-9 mRNA was altered in most cancers. The Siglec-9 mRNA expression level was correlated with the survival of patients. It was also correlated with other prognostic factors such as immune checkpoint, mismatch repair (MMR), DNA methyltransferase (DNMT), tumor mutation burden (TMB), and microsatellite instability (MSI). In addition, Siglec-9 expression was strongly correlated with the tumor immune microenvironment including infiltration of immune cells, immune score, immune subtypes, and immune checkpoint expression. Moreover, the correlation between Siglec-9 expression and prognosis or immune cell infiltration in LGG was further verified using the dataset from the Chinese Glioma Genome Atlas (CGGA).

The present study reported the expression pattern of Siglec-9 and its relationship with prognosis and tumor immune microenvironment in pan-cancer. It uncovered the prognostic value of Siglec-9 in some cancers including LGG and suggested that Siglec-9 can be a novel biomarker and a potential target for cancer immunotherapy.

## Materials and Methods

### Data Collection

The mRNA expression, copy number alteration, and clinical information of more than 30 cancer types were obtained from The Cancer Genome Atlas (TCGA). The mRNA expression data from normal tissue sites across nearly 1,000 people were obtained from the Genotype-Tissue Expression (GTEx) to supplement the normal tissue mRNA sequencing data lacking in TCGA. Siglec-9 mRNA expression data of tumor cell lines were obtained from the Broad Institute Cancer Cell Line Encyclopedia (CCLE). The mRNA expression data and clinical information of glioma were obtained from the Chinese Glioma Genome Atlas (CGGA) to verify the result in TCGA-LGG. We used the “mRNAseq_693” dataset (Wang et al., 2015; Liu et al., 2018). WHO grade II and WHO grade III cases were defined as LGG to explore the role of Siglec-9 in LGG (Liu et al., 2018).

### Correlation of Siglec-9 Expression to Tumor Clinical Characteristics

GEPIA2 (http://gepia2.cancer-pku.cn/#index) (Tang et al., 2019) was used to analyze the correlation between tumor stages and Siglec-9 mRNA expression using “major stage” and “log2 (TPM + 1) for log-scale”. The association between Siglec-9 mRNA expression and overall survival (OS), disease-specific survival (DSS), disease-free interval (DFI), and progression-free interval (PFI) depended on TCGA databases and was analyzed on the DriverDBv3 (http://driverdb.tms.cmu.edu.tw/) (Liu et al., 2020) with mean of Siglec-9 expression as cutoff value. The online database UALCAN network (http://ualcan.path.uab.edu/) (Chandrashekar et al., 2017) was also used to verify the OS analysis with default settings. Siglec-9 expression in diverse molecular subtypes was analyzed on The Integrated Repository Portal for Tumor-Immune System Interactions (TISIDB, http://cis.hku.hk/TISIDB/index.php) (Ru et al., 2019).

### Correlation of Siglec-9 Expression to Immunological Characteristics

TIMER (https://cistrome.shinyapps.io/timer/) (Li et al., 2017) and TIMER2.0 (http://timer.comp-genomics.org/) (Li et al., 2020) were used to assess the correlation between the Siglec-9 expression and immune cell infiltration for TCGA tumors and CGGA-LGG dataset with TIMER score and CIBERSORT score (Newman et al., 2015) using immune infiltrates query function and estimation function. The results of estimation were visualized by R package “psych”. TISIDB, a user-friendly web instrument to explore comprehensive investigation of tumor-associated immunity, was used to analyze the association between Siglec-9 mRNA expression and tumor immune subtypes, and specific types of immune cell infiltration. We estimated Siglec-9 expression in diverse molecular subtypes, and immune subtypes involving C1 (wound healing), C2 (IFN-γ dominant), C3 (inflammatory), C4 (lymphocyte deplete), C5 (immunologically quiet), and C6 (TGF-β dominant) subtypes. Specific types of immune cell infiltration of 28 TIL types were inferred by using gene set variation analysis (GSVA) based on the Siglec-9 expression profile on TISIDB.

### Gene Set Enrichment Analysis

GSEA based on WEB-based Gene SeT AnaLysis Toolkit (WebGestalt, http://www.webgestalt.org/) (Liao et al., 2019) was performed with the TCGA-LGG dataset on LinkedOmics (Vasaikar et al., 2018) (http://www.linkedomics.org/login.php), a publicly available portal that includes multi-omics data from all 32 TCGA Cancer types. CGGA mRNA expression data were downloaded and LGG cases were selected to be analyzed with GSEA on WebGestalt. The parameters for the enrichment analysis were as follows: minimum number of IDs in the category: three; maximum number of IDs in the category: 2000; significance level: Top 25; number of permutation: 1,000. The top ten positive related categories are shown in the main figure.

### Statistical Analysis

The expression of Siglec-9 in different tissue was used by Kruskal–Wallis test, and between tumor tissues and normal tissues were estimated by *t*-test. Additionally, the expression of Siglec-9 in different grades of glioma was utilized by *t*-test and ANOVA test. Kaplan–Meier curves were visualized to compare the survival patients stratified based on different levels of expression of Siglec-9. The relationship between Siglec-9 and TMB (tumor mutation burden), MSI (microsatellite instability), MMR gene mutation, immune checkpoints, DNMT, immune score, stromal score, ESTIMATE score, and immune cells was evaluated by Pearson and Spearman correlation analyses. *p* < 0.05 was recorded as statistically significant for all analyses unless otherwise specified.

## Results

### The Expression Pattern of Siglec-9 mRNA in Human Cancers

The expression of Siglec-9 mRNA in normal tissue was firstly analyzed using the GTEx database. It showed that the level of Siglec-9 expressions was highest in tissues of blood, spleen, and lung and the lowest in muscle and bone marrow among all types of tissues assessed (Kruskal–Wallis test, *p* = 0) (Supplementary Figure S1).

**FIGURE 1:**
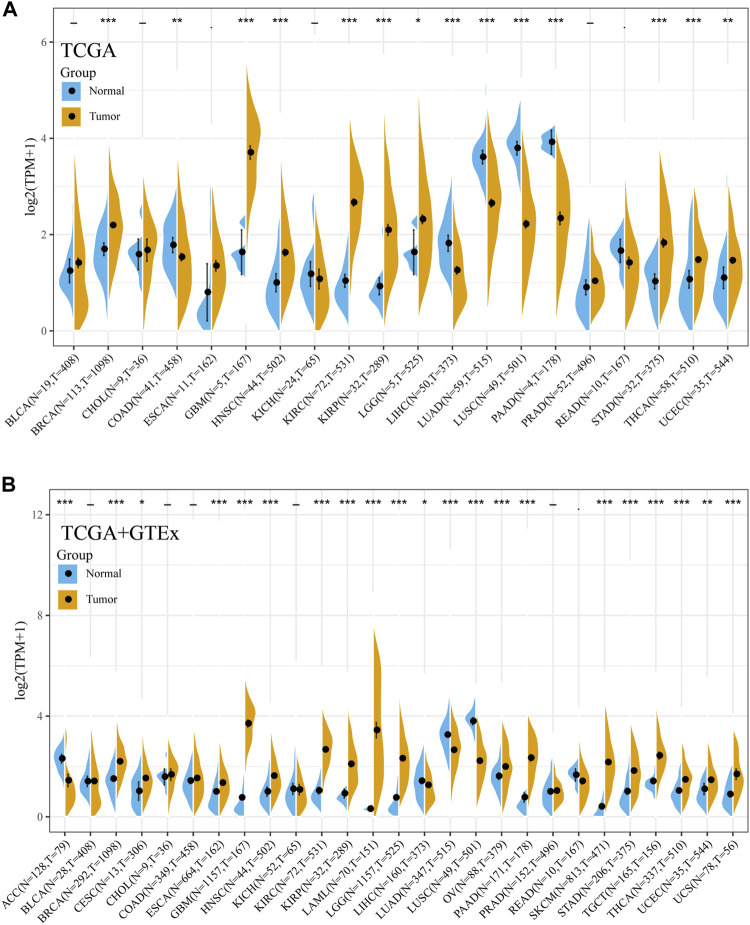
The mRNA expression of Siglec-9 in various cancers according to The Cancer Genome Atlas (TCGA) and Genotype-Tissue Expression (GTEx). **(A)** Siglec-9 expression in pan-cancer relied on TCGA. **(B)** Sigelc-9 expression in pan-cancers based on TCGA and the normal samples were enriched with the GTEx database. (*t*-test, *p* < 0.05 was considered to be significant, *p < 0.05, **p < 0.01, ***p < 0.001. N: Normal tissue; T: Tumor tissue.)

Then, the expression of Siglec-9 in tumor and normal samples from various tissue types was assessed using TCGA database. Differential expression analysis was performed with normal tissues used as control. Siglec-9 was upregulated in tumor tissues from cancers including breast invasive carcinoma (BRCA), glioblastoma multiforme (GBM), head and neck squamous cell carcinoma (HNSC), kidney renal clear cell carcinoma (KIRC), kidney renal papillary cell carcinoma (KIRP), brain lower-grade glioma (LGG), stomach adenocarcinoma (STAD), thyroid carcinoma (THCA), and uterine corpus endometrial carcinoma (UCEC) (Figure 1A). Siglec-9 was downregulated in cancers including colon adenocarcinoma (COAD), liver hepatocellular carcinoma (LIHC), lung adenocarcinoma (LUAD), lung squamous cell carcinoma (LUSC), and pancreatic adenocarcinoma (PAAD) (Figure 1A).

For the tumors lacking normal sample controls in TCGA, the corresponding normal samples from GTEx databases were included for further differential expression analysis. The result showed that Siglec-9 was upregulated in BRCA, cervical squamous cell carcinoma and endocervical adenocarcinoma (CESC), esophageal carcinoma (ESCA), acute myeloid leukemia (LAML), LGG, ovarian serous cystadenocarcinoma (OV), pancreatic adenocarcinoma (PAAD), skin cutaneous melanoma (SKCM), STAD, testicular germ cell tumors (TGCT), THCA, UCEC, and uterine carcinosarcoma (UCS), and was downregulated in adrenocortical carcinoma (ACC) (Figure 1B).

**FIGURE 2:**
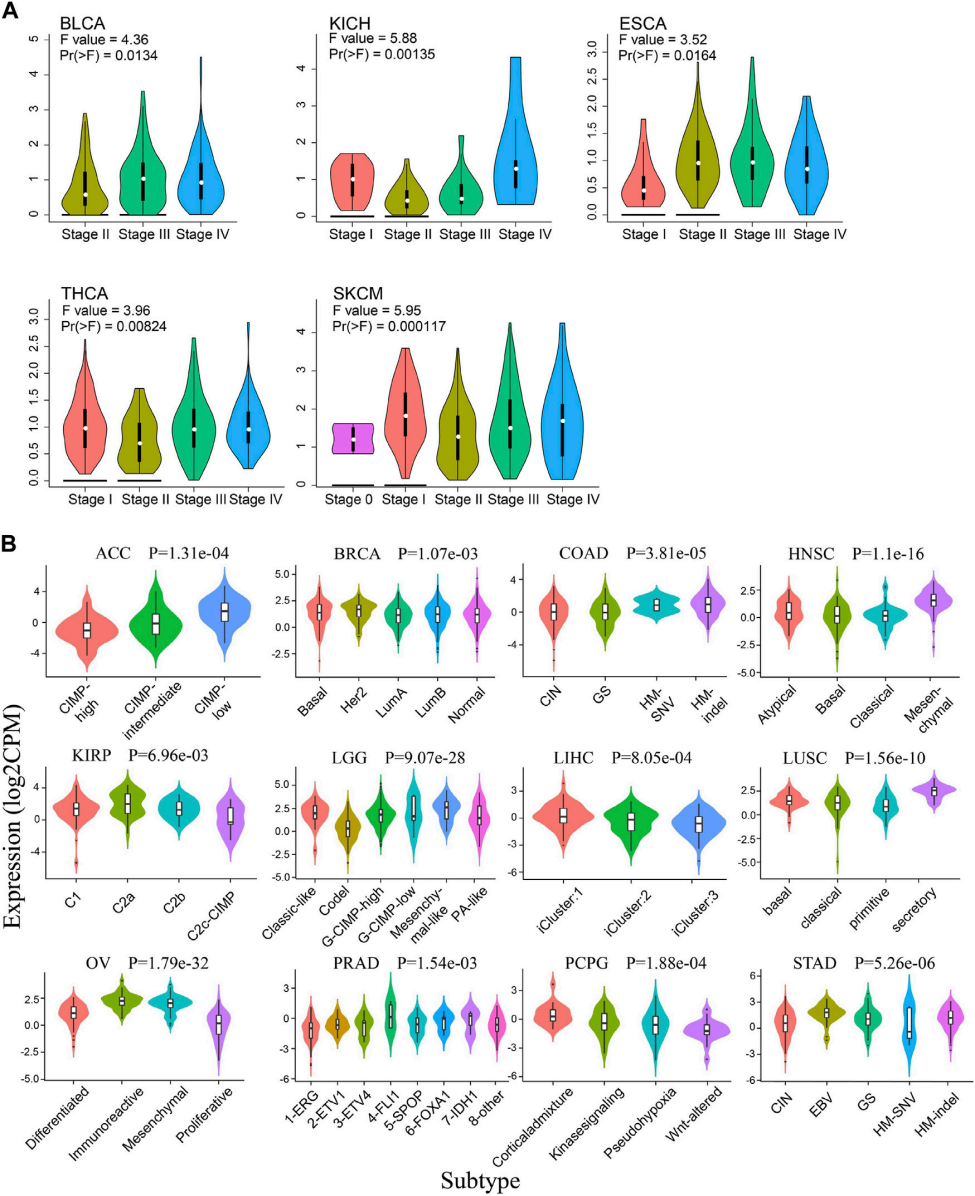
The correlation between Siglec-9 mRNA expression and tumor stages and molecular subtypes. **(A)** The correlation between Siglec-9 expression and tumor stage in various cancers obtained from Gene Expression Profiling Interactive Analysis2.0 (GEPIA2.0). Tumors with a significant result were shown. [Vertical axis: log2 (TPM + 1); one-way ANOVA, Pr (>F) < 0.05 was considered to be significant. The larger the F value and the smaller the Pr (>F) value, the greater the difference in characteristics between groups.] **(B)** The correlation between Siglec-9 expression and different molecular subtypes from Integrated Repository Portal For Tumor-Immune System Interactions (TISIDB). Tumors with significant result were shown. (Kruskal–Wallis test, *p* < 0.05 was considered to be significant. CPM: count-per-million.)

In addition, the expression pattern of Siglec-9 in the tumors of different clinal stages and molecular subtypes in pan-cancer was also investigated. It showed that Siglec-9 expression was significantly different among clinal stages in bladder urothelial carcinoma (BLCA), ESCA, KICH, THCA, and SKCM (Figure 2A). The different expression pattern was also observed among molecular subtypes in ACC, BRCA, COAD, HNSC, KIRP, LGG, LIHC, LUSC, OV, PCPG, PRAD, and STAD (Figure 2B). No different expression was found among tumor stages or molecular subtypes in other tumors such as ACC, BRCA, and CESC (Supplementary Figure S2A **)**.

### Correlation Analysis of Siglec-9 Expression and Prognostic Value in Pan-Cancer

**FIGURE 3:**
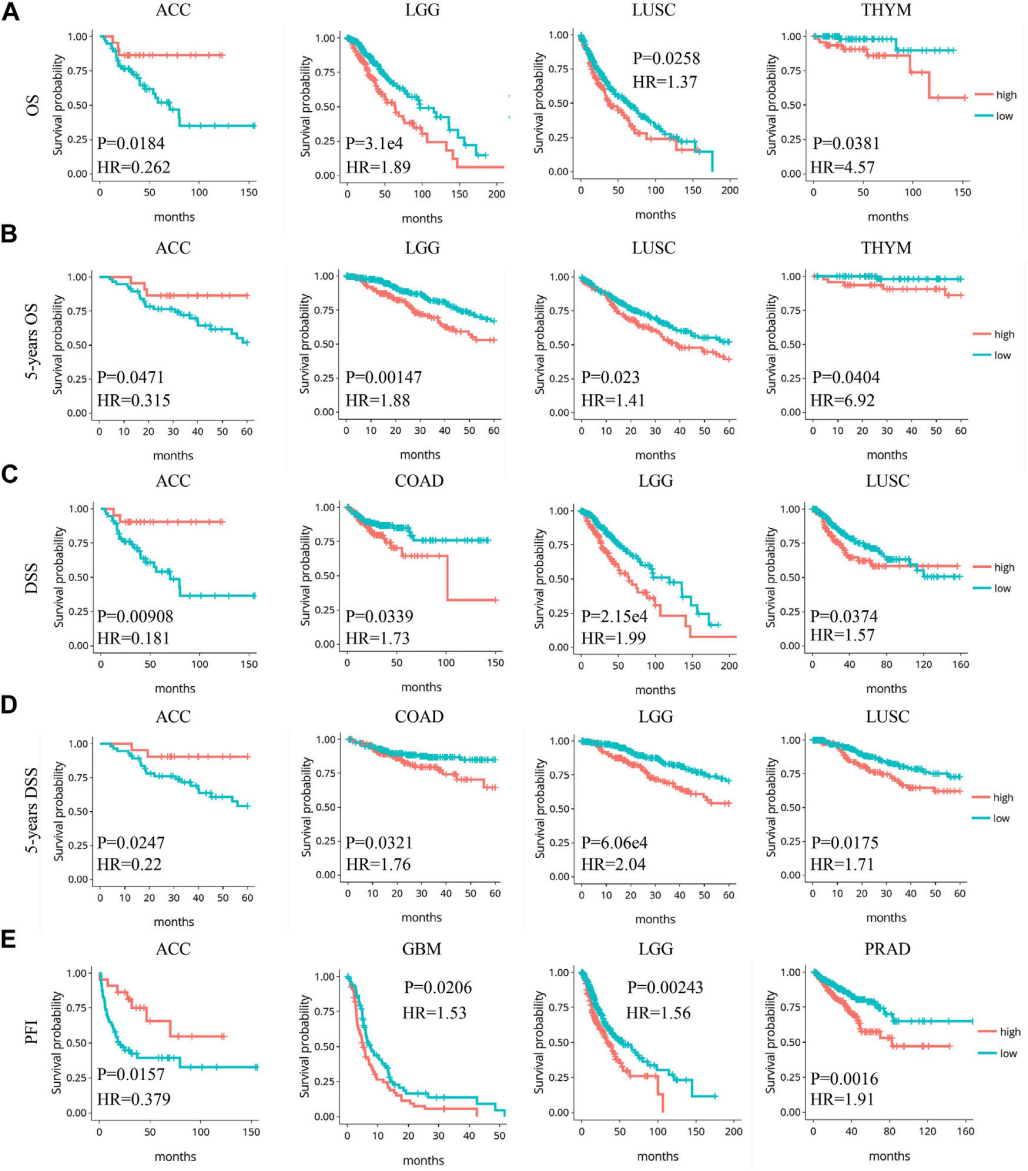
Significant correlation between Siglec-9 expression and prognostic value in pan-cancer relied on DriverDBv3. **(A)** Expression of Siglec-9 was associated with overall survival (OS) in LGG, ACC, LUSC, and THYM. **(B)** Expression of Siglec-9 was associated with 5-year OS in LGG, ACC, LUSC, and THYM. **(C)** Expression of Siglec-9 was associated with disease-specific survival (DSS) in ACC, COAD, LGG, LUSC. **(D)** Expression of Siglec-9 was associated with 5-year DSS in ACC, COAD, LGG, and LUSC. **(E)** Expression of Siglec-9 was associated with progression-free interval (PFI) in ACC, GBM, LGG, and PRAD (*p* < 0.05 was considered significant. HR: hazard ratio).

The prognostic value of Siglec-9 expression was assessed by investigating the relationship between Siglec-9 expression and clinical characteristics including OS, DSS, DFI, and PFI using DriverDBv3. Higher Siglec-9 expression level was found to be associated with shorter OS and 5-year OS (HR > 1) in LUSC, THYM, and LGG, but longer OS and 5-year OS (HR < 1) in ACC (Table 1; Figures 3A,B; Supplementary Figure S3A). DSS analysis was performed to further verify these correlations and found similar results (Figures 3C,D; Supplementary Table S1 **)**. High Siglec-9 expression correlated with the poor outcome of PFI in GBM, PRAD, and LGG, while it correlated with a good outcome of PFI in ACC (Supplementary Table S2; Figure 3E; Supplementary Figure S3B). DFI analysis found that increased Siglec-9 expression was associated with shorter OS and 5-year survival in ESCA only (Supplementary Table S3; Supplementary Figure S3C **)**. OS analysis was also performed using UALCAN for verification. It showed that Siglec-9 was a poor prognostic factor in LGG, SKCM, UVM, and a good one in READ (Supplementary Table S4). A significant correlation of Siglec-9 with the prognosis in LGG was observed from both DriverDBv3 and UALCAN analysis (Table 1; Supplementary Table S4).

**TABLE 1:** Overall survival analysis of TCGA tumors by DriverDBv3.

Cancer type	Overall survival		5-year survival		Number of high expressed example	Number of low expressed example
	Log-rank *p*-value	HR	Log-rank *p*-value	HR		
GBM	0.0629	1.41	0.0761	1.39	68	85
OV	0.146	1.22	0.186	1.22	144	228
LUAD	0.508	0.902	0.281	0.842	200	300
LUSC	0.0258 (*)	1.37	0.0231 (*)	1.41	174	320
PRAD	0.546	0.661	0.238	0.296	192	303
UCEC	0.388	0.82	0.33	0.79	185	356
BLCA	0.725	1.06	0.768	1.05	119	287
TGCT	0.169	4.63	0.169	4.63	40	94
ESCA	0.534	0.846	0.534	0.846	58	103
PAAD	0.662	1.1	0.578	1.13	72	104
KIRP	0.826	1.08	0.571	0.806	86	199
CESC	0.925	1.02	0.987	0.996	102	189
LIHC	0.519	1.12	0.756	1.06	124	241
SARC	0.262	0.769	0.339	0.787	80	179
BRCA	0.493	0.886	0.757	1.07	422	654
THYM	0.0381 (*)	4.57	0.0404 (*)	6.92	47	71
MESO	0.735	0.92	0.735	0.92	33	51
COAD	0.0524	1.49	0.0792	1.46	155	283
STAD	0.972	0.994	0.952	1.01	136	217
CHOL	0.151	0.49	0.217	0.535	15	21
KIRC	0.176	1.23	0.306	1.18	219	309
THCA	0.934	1.04	0.579	0.721	179	322
HNSC	0.687	1.06	0.81	1.04	177	322
LAML	0.0595	1.54	0.0595	1.54	43	87
READ	0.132	1.86	0.132	1.86	59	100
SKCM	0.344	0.691	0.344	0.691	38	64
LGG	0.00031 (***)	1.89	0.00147 (**)	1.88	176	330
DLBC	0.221	0.371	0.221	0.371	21	26
KICH	0.941	0.943	0.905	1.1	18	46
UCS	0.245	1.52	0.32	1.45	19	36
ACC	0.0184 (*)	0.262	0.0471 (*)	0.315	22	57
PCPG	0.938	1.07	0.786	1.28	62	116
UVM	0.184	1.75	0.184	1.75	28	52

*p < 0.05; **p < 0.01; ***p < 0.001.

### Correlation Between Siglec-9 Expression and Tumor Immune Microenvironment

**FIGURE 4:**
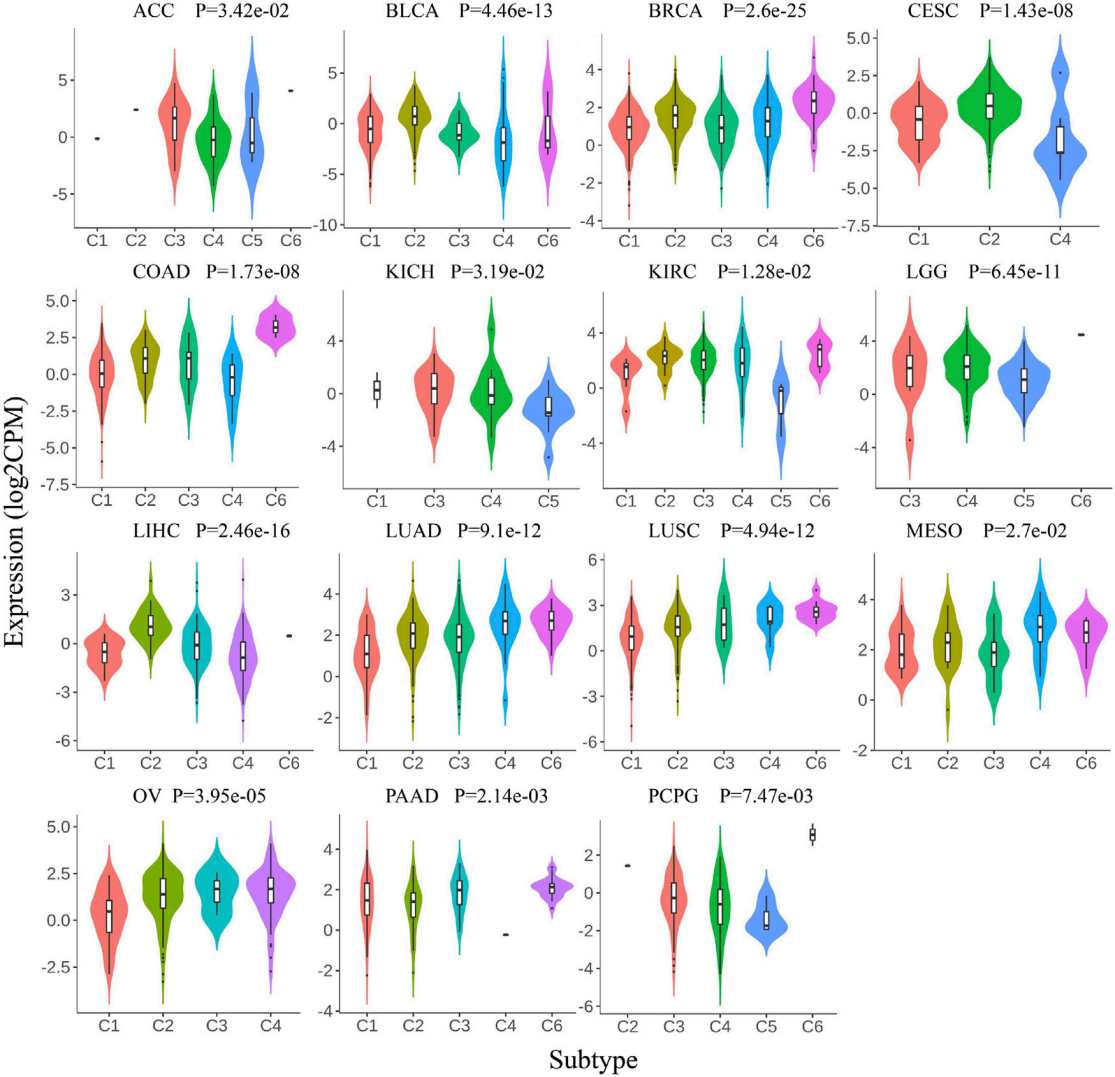
Correlation between Siglec-9 mRNA expression and tumor immune subtypes depended on TISIDB. Siglec-9 mRNA expression was related to immune subtypes in various cancers (top 15 tumors were shown). Immune subtypes included: C1, wound healing; C2, IFN-γ dominant; C3, inflammatory; C4, lymphocyte depletes; C5, immunologically quiet; and C6, TGF-β dominant. (Kruskal–Wallis test, *p* < 0.05 was considered to be significant. CPM: count-per-million.)

Previous research has identified six molecular immune subtypes containing wound healing (C1), IFN-γ dominant (C2), inflammatory (C3), lymphocyte depletes (C4), immunologically quiet (C5), and TGF-β dominant (C6) subtypes, which were related to tumor molecular characterization and prognosis of patients (Thorsson et al., 2018). We analyzed the expression pattern of Siglec-9 in six immune subtypes in TCGA pan-cancer. The Siglec-9 was differentially expressed in different immune subtypes in ACC, BLCA, BRCA, CESC, COAD, KICH, KIRC, LGG, LUSC, LIHC, LUAD, Mesothelioma (MESO), OV, PAAD, PCPG, PRAD, READ, SARC, SKCM, STAD, TGCT, THCA, UCEC, UCS, and UVM (Figure 4; Supplementary Figure S4A). No difference among different immune subtypes was observed in other tumors including CHOL, ESCA, GBM, HNSC, and KIRP (Supplementary Figure S4B).

**FIGURE 5:**
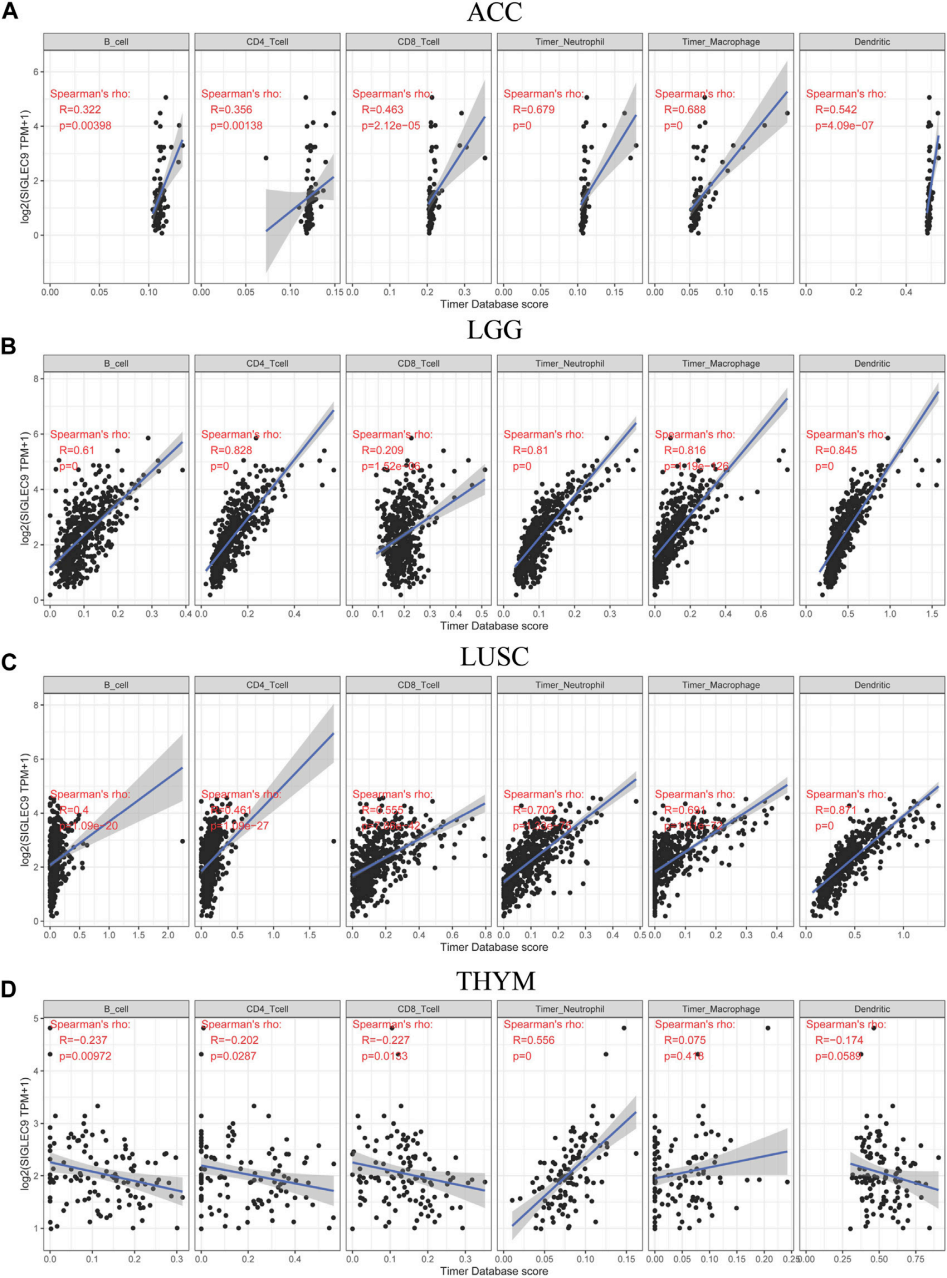
Correlation between Siglec-9 mRNA expression and immune cell infiltration (B cell, CD4^+^ T cell, CD8^+^ T cell, neutrophil, macrophage, and dendritic cell) in ACC **(A)**, LGG **(B)**, LUSC **(C)**, and THYM **(D)** according to TIMER. (Spearman correlation, *p* < 0.05 was considered significant. The larger the *R*-value, the greater the correlation.)

**FIGURE 6:**
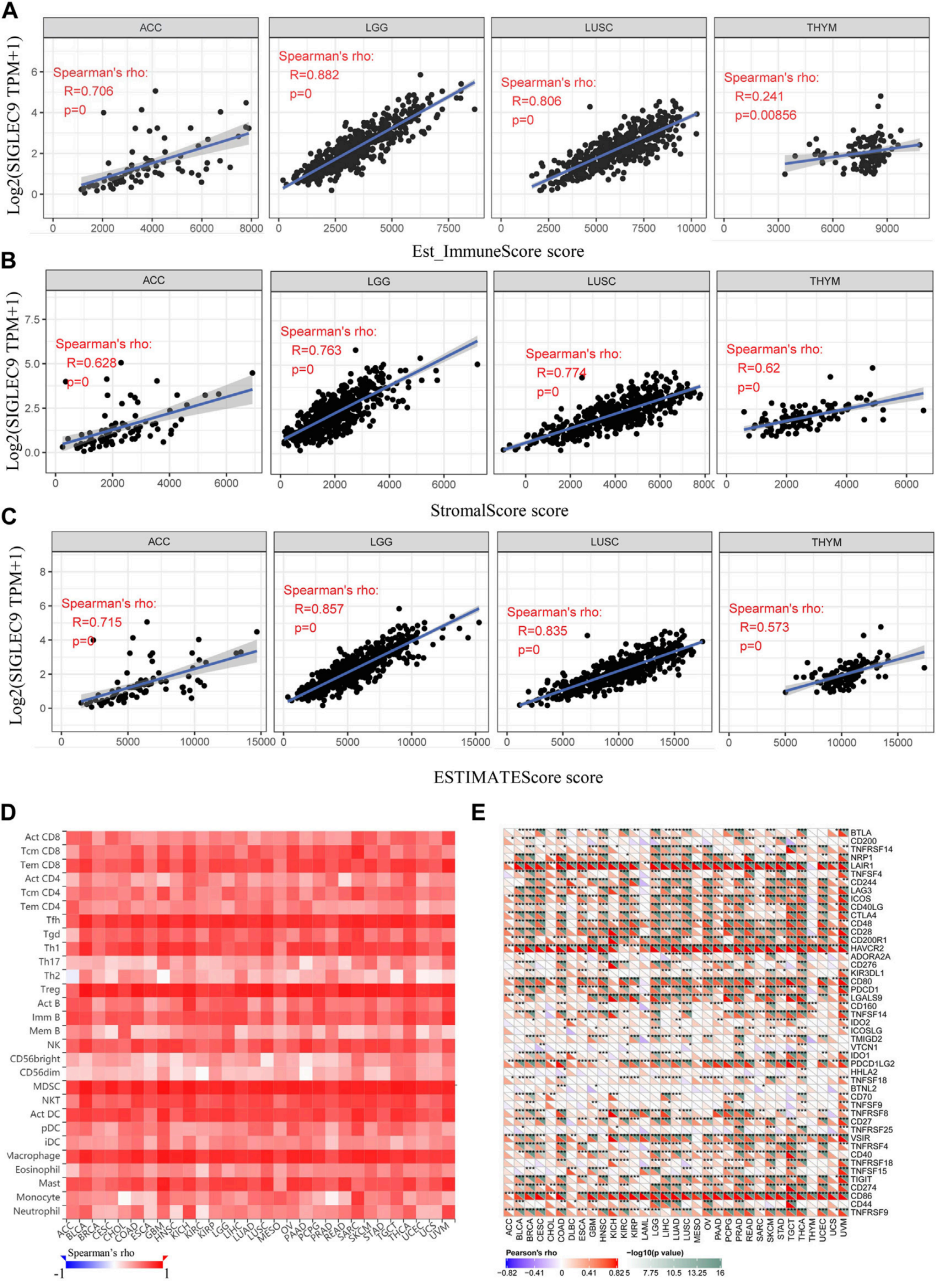
The relationship between Siglec-9 mRNA expression and immune score, stromal score, ESTIMATE score, specific immune cell infiltration, and immune checkpoints. **(A)** Correlation between Siglec-9 mRNA expression and Immune Score in ACC, LGG, LUSC, and THYM. **(B)** Correlation between Siglec-9 mRNA expression and Stromal Score in ACC, LGG, LUSC, and THYM. **(C)** Correlation between Siglec-9 mRNA expression and ESTIMATE score in ACC, LGG, LUSC, and THYM. **(D)** Correlation between immune cells and Siglec-9 mRNA expression according to TISIDB. **(E)** Correlation between the immune checkpoint and Siglec-9 mRNA expression. **(A–D)** Spearman correlation; **(E)** Pearson’s correlation (*p* < 0.05 was considered significant).

Then, the association between Siglec-9 expression and immune cell infiltration in tumor tissues was analyzed using TIMER. A positive correlation between Siglec-9 expression and the infiltration of B cells, CD4^+^ T cells, CD8^+^ T cells, neutrophils, macrophages, and dendritic cells was observed in most TCGA tumors including LGG, ACC, and LUSC (Figures 5A–C; Supplementary Figure S5). In THYM, a negative correlation was observed in B-cell, CD4^+^ T-cell, and CD8^+^ T-cell infiltration and a positive correlation was observed in neutrophil infiltration (Figure 5D **)**. Besides, Siglec-9 expression was negatively correlated with CD8^+^ T cells in GBM, with B cells in STAD and UVM, and with macrophages in DLBC (Supplementary Figure S5). ESTIMATE analysis was applied to further investigate the correlation between Siglec-9 expression and the tumor microenvironment. A strong positive correlation was observed in immune scores (Supplementary Figure S6), stromal scores (Supplementary Figure S7), and ESTIMATE scores (Supplementary Figure S8) in all 33 tumor types in TCGA including ACC, LGG, LUSC, and THYM (Figures 6A–C). To explore the association between Siglec-9 expression and specific types of immune cell infiltration, the relative abundance of 28 TIL types was inferred by using gene set variation analysis (GSVA) based on the Siglec-9 expression profile on TISIDB. The result showed that Siglec-9 expression was strongly correlated with follicular helper T cells (Tfh), regulatory T cells (Treg), myeloid-derived suppressor cells (MDSC), and macrophages across 33 TCGA tumors (Figure 6D). Moreover, the correlation between Siglec-9 expression and the expression of 47 immune checkpoint genes was also analyzed. Siglec-9 was positively correlated with immune checkpoints including LAIR1, HAVCR2, CD80, PDCD1 (PD1), PDCD1LG2 (PD-L2), VSIR, and CD86 across most TCGA tumors (Figure 6E). Siglec-9 had a low correlation with immune checkpoints in tumors including ACC, CHOL, DLBC, LAML, MESO, THYM, and UCS. In particular, LAIR1, HAVCR2, LGALS9, and CD86 expression were strongly positively correlated with Siglec-9 expression, and only CD200 expression was negatively correlated with Siglec-9 expression in LGG. In LUSC, Siglec-9 expression was strongly positively correlated with LAIR1, HAVCR2, and CD86, and negatively correlated with VTCN1 and TNFRSF18.

### Correlation Between Siglec-9 Expression and Tumor Mutation Burden, Microsatellite Instability, DNA Mismatch Repair Genes, and DNA Methyltransferase

**FIGURE 7:**
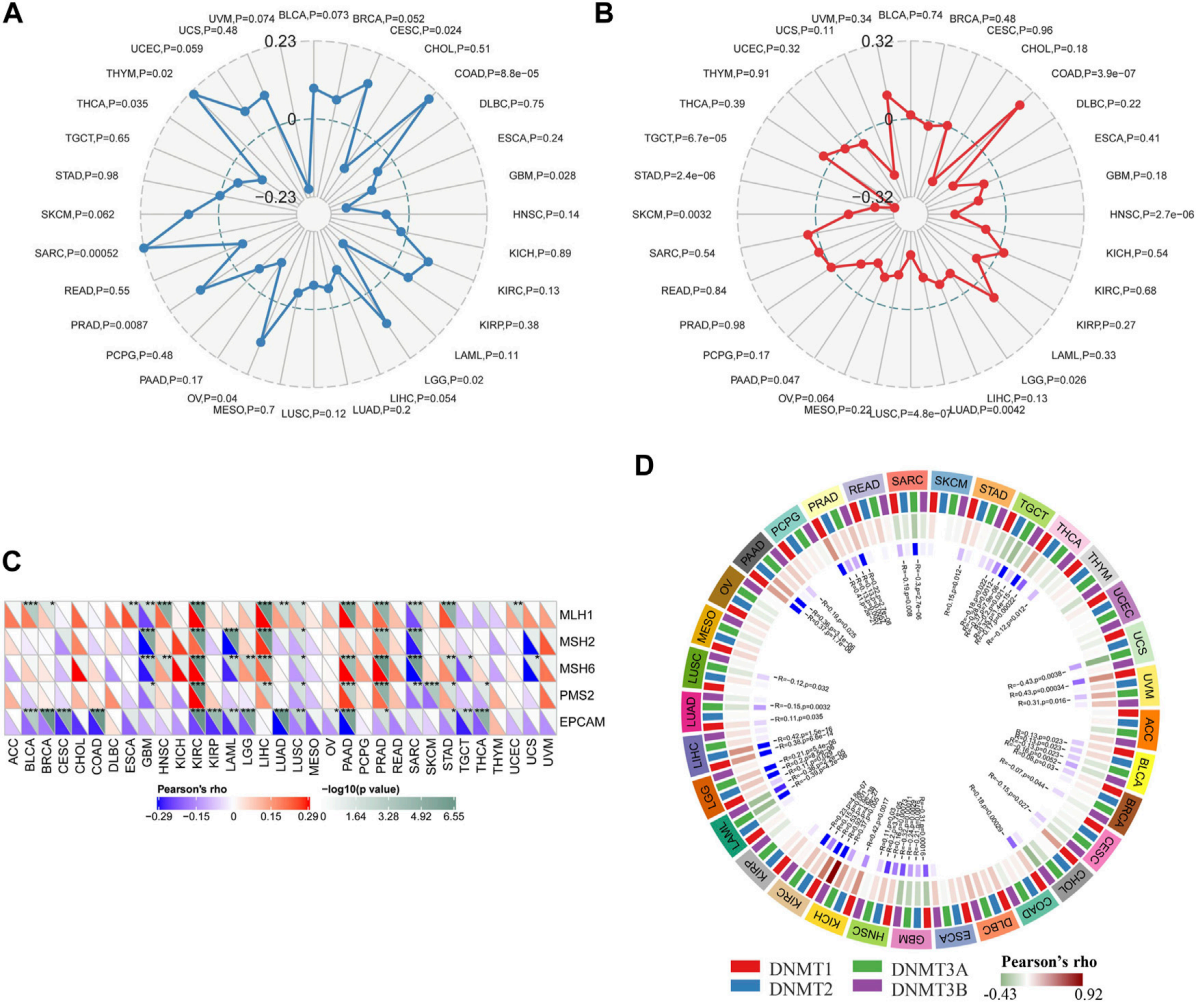
Correlation between Siglec-9 mRNA expression and tumor mutation burden (TMB), microsatellite instability (MSI), DNA methyltransferase (DNMT), and mismatch repair (MMR) in pan-cancer. **(A)** Relationship between Siglec-9 expression and TMB. **(B)** Relationship between Siglec-9 expression and MSI. **(C)** Correlation between Siglec-9 expression and MMR. **(D)** Correlation between Siglec-9 expression and DNMT (DNMT1 was red, DNMT2 was blue, DNMT3 was green, DNMT4 was purple). (Pearson’s correlation, *p* < 0.05 was considered significant, *p < 0.05, **p < 0.01, ***p < 0.001.)

TMB, MSI, DNA MMR, and DNA methylation were considered immune-related and prognosis-related factors in tumors (Duffy and Crown, 2019). Therefore, we analyzed the correlation between the Siglec-9 expression and TMB, MSI, DNA MMR genes, and DNMT. Siglec-9 expression was found to be positively correlated with TMB in CESC, COAD, LGG, OV, PRAD, SARC, and THYM and negatively correlated with TMB in GBM and THCA (Figure 7A). Siglec-9 expression was negatively related to MSI in HNSC, LGG, LUAD, LUSC, PAAD, SKCM, STAD, and TGCT and positively related to MSI in COAD (Figure 7B). Five DNA MMR genes (MLH1, MSH2, MSH6, PMS2, and EpCAM) were included for analyzing the association between MMRs and Siglec-9. MLH1 was positively correlated with Siglec-9 in BLCA, BRCA, ESCA, HNSC, KIRC, LIHC, LUAD, LUSC, PAAD, PRAD, STAD, UCEC, and negatively correlated in GBM and SARC (Figure 7C). MSH2 had a positive correlation with Siglec-9 in KIRC, LIHC, and PRAD, and had a negative correlation in (Figure 7C). MSH6 showed a positive association with Siglec-9 in HNSC, KIRC, LGG, LIHC, PAAD, PRAD, and STAD, and a negative association in GBM, LAML, LUSC, SARC, TGCT, and UCS (Figure 7C). PMS2 was positively correlated with Siglec-9 in KIRC, LIHC, PAAD, PRAD, and STAD, and negatively correlated in GBM, LUSC, SARC, SKCM, and THCA (Figure 7C). Siglec-9 was negatively correlated to EpCAM in most tumors including BLCA, BRCA, CESC, CHOL, COAD, KIRC, KIRP, LAML, LGG, LUAD, LUSC, OV, PAAD, PRAD, STAD, TGCT, and THCA (Figure 7C). Collectively, Siglec-9 had a significant association with MMRs in KIRC, PAAD, and PRAD (Figure 7C).

DNA methyltransferase1 (DNMT1), DNA methyltransferase2 (DNMT2), DNA methyltransferase3 (DNMT3), and DNA methyltransferase3B (DNMT3B) were included for the analysis of the correlation between Siglec-9 expression and DNA methyltransferases. It showed that Siglec-9 expression was correlated with DNA methylation in various cancers, especially in BLCA, GBM, HNSC, KICH, KIRC, LGG, PAAD, PRAD, TGCT, and THCA (Figure 7D). In particular, Siglec-9 was positively correlated with DNMT1 (Pearson’s rho = 0.11, *p* = 0.029), DNMT2 (Pearson’s rho = 0.2, *p* = 8.5 e-6), and DNMT3 (Pearson’s rho = 0.21, *p* = 5.4 e-6). There was no significant correlation or less correlation between Siglec-9 expression and DNA methyltransferases in ACC, LUSC, and THYM.

### Further Validation of Siglec9 Expression Pattern in Brain Lower-Grade Glioma

**FIGURE 8:**
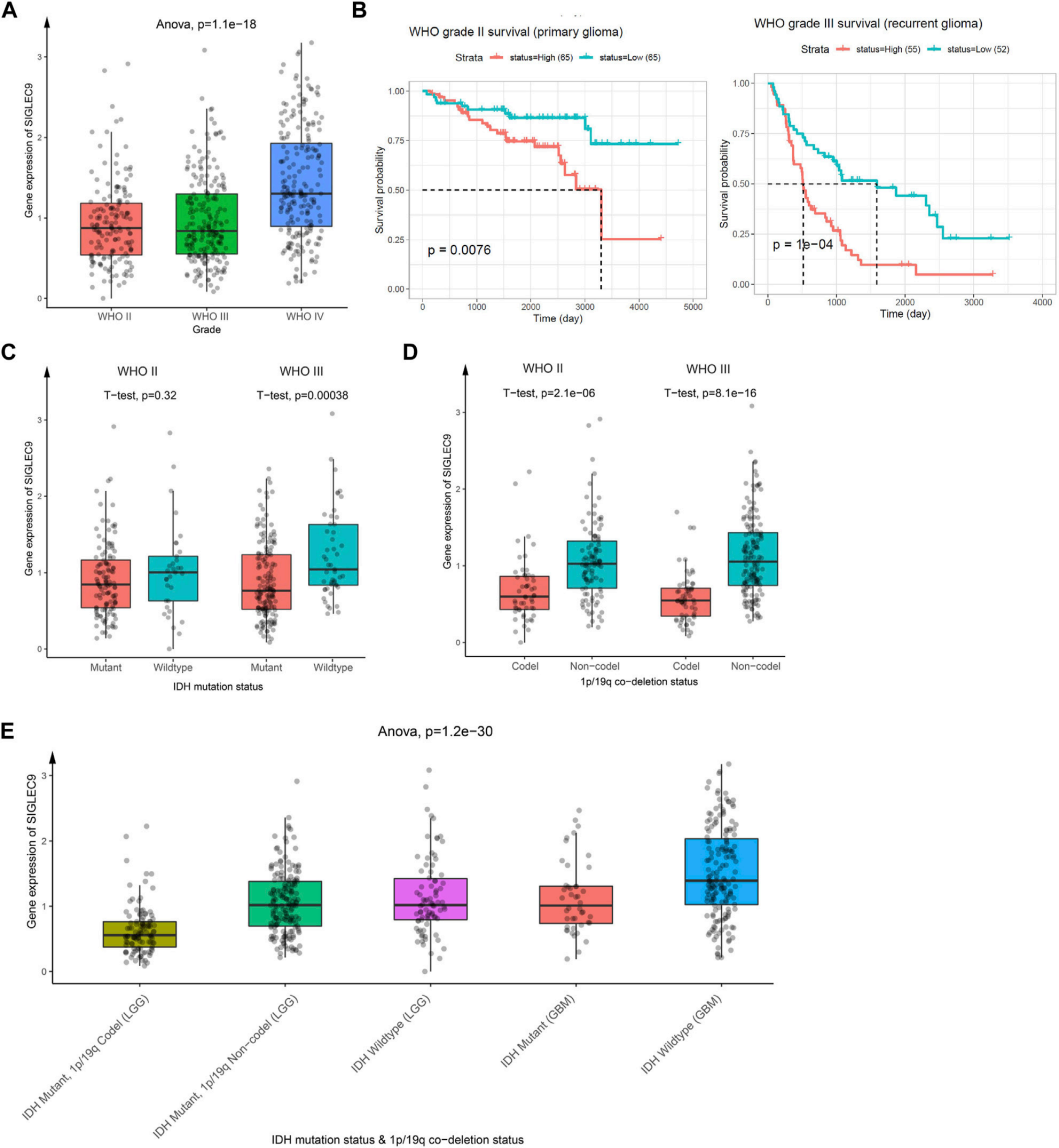
The relationship between Siglec-9 mRNA expression and clinical characteristics in brain lower-grade glioma (LGG). **(A)** Siglec-9 mRNA expression in different glioma grades. **(B)** Expression of Siglec-9 was related to OS in WHO grade II (primary glioma) and WHO grade III (recurrent glioma). **(C)** Different Siglec-9 expression in WHO grade II and WHO grade III depended on IDH mutation status. **(D)** Different Siglec-9 expression in WHO grade II and WHO grade III depended on 1p/19q co-deletion status. **(E)** Different Siglec-9 expression in WHO grade II and WHO grade III depended on IDH mutation status combined with 1p/19q co-deletion status. (Statistical methods are shown in the figures. *P* < 0.05 was considered significant. IDH: isocitrate dehydrogenase.)

**FIGURE 9:**
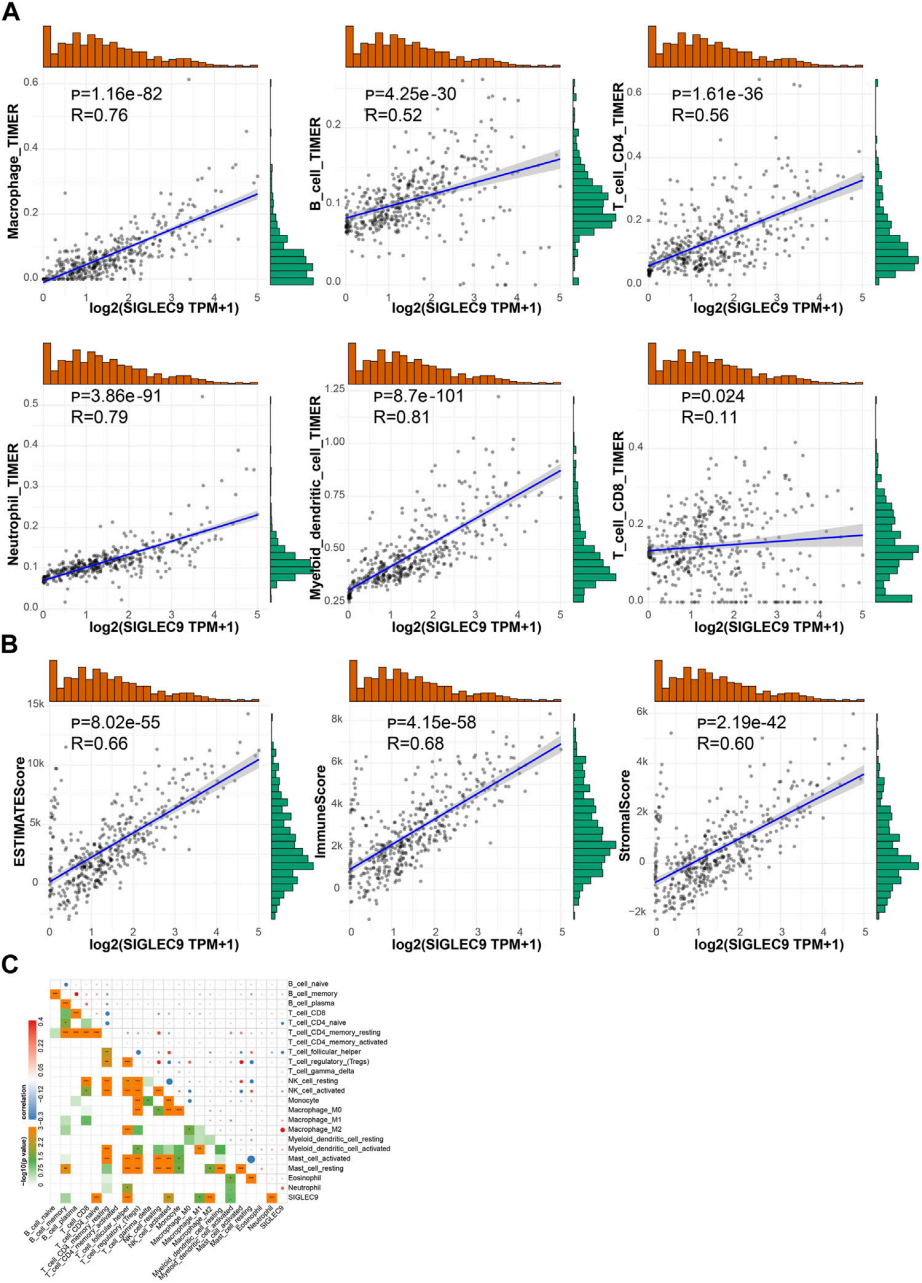
Correlation between Siglc-9 expression and immune cell infiltration and immune score, stromal score, and ESTIMATE score in brain lower-grade glioma (LGG). **(A)** Relationship between Siglec-9 expression and immune cell infiltration (macrophage, B cell, CD4^+^ T cell, neutrophil, dendritic cell, and CD8^+^ T cell). **(B)** Correlation between Siglec-9 mRNA expression and ESTIMATE score, immune score, and stromal score. **(C)** Correlation between Siglec-9 expression and specific immune cell infiltration. (Spearman correlation, *p* < 0.05 was considered significant, *p < 0.05, **p < 0.01, ***p < 0.001. The larger the *R*-value, the greater the correlation.)

The analysis above showed that Siglec9 expression was correlated with the prognosis of patients, especially of ACC and LGG patients (Figure 3). Due to the larger sample size, similar survival pattern to other tumors, and closer association with the tumor immune microenvironment, TMB, MSI, MMRs, and DNMTs, we selected LGG for further analysis. Data from the Chinese Glioma Genome Atlas (CGGA) was employed for further verification. Expression of Siglec-9 in different grades of glioma (WHO II, WHO III, and WHO IV) was analyzed. The results showed that Siglec-9 expression was higher in WHO IV (GBM) than that in WHO II and WHO III (LGG) (Figure 8A). OS analysis showed that high expression of Siglec-9 was correlated with poor prognosis in primary WHO grade II and recurrent WHO grade III glioma (Figure 8B). There was no significant correlation between OS and Siglec-9 expression in recurrent WHO grade II (Supplementary Figure S9A) and primary WHO grade III glioma (Supplementary Figure S9B). In addition, Siglec-9 expression in IDH mutation and 1p/19q co-deletion status subtypes was analyzed, because IDH mutation status and 1p/19q co-deletion status were beneficial to diagnosis, prognosis prediction, and treatment of glioma (Aoki et al., 2018). No difference between IDH mutant and wild type in WHO grade II was observed, and higher Siglec-9 expression was observed in wild type compared to IDH mutant in WHO grade III (Figure 8C). Besides, Siglec-9 expression was significantly higher in the none 1p/19q co-deletion than in the 1p/19q co-deletion subtype in both WHO grade II and WHO grade III (Figure 8D), which coincided with the former result of low expression of Siglec-9 in Codel subtype in the TCGA-LGG (Figure 2B). Subsequently, Siglec-9 expression in subtypes that combined IDH mutation with 1p/19q was also analyzed in LGG and GBM. The result showed that Siglec-9 was significantly differentially expressed among these subtypes (Figure 8E). Then, TIMER was used to assess the correlation between Siglec-9 expression and immune cell infiltration in CGGA-LGG. Similar to TCGA-LGG, Siglec-9 expression was positively correlated with B cells, CD4^+^ T cells, neutrophils, macrophages, and myeloid dendritic cells except for CD8^+^ T cells according to TIMER immune score in CGGA-LGG (Figure 9A). A strong correlation was observed between Siglec-9 expression and immune score, stromal score, and ESTIMATE score (Figure 9B). Analysis of correlation between Siglec-9 expression and specific immune cells indicated that high Siglec-9 expression was accompanied by high infiltration level of M1/2 macrophages and neutrophils, and low infiltration level of activated NK cells, Tfh, and naïve CD4^+^ T cells (Figure 9C).

**FIGURE 10:**
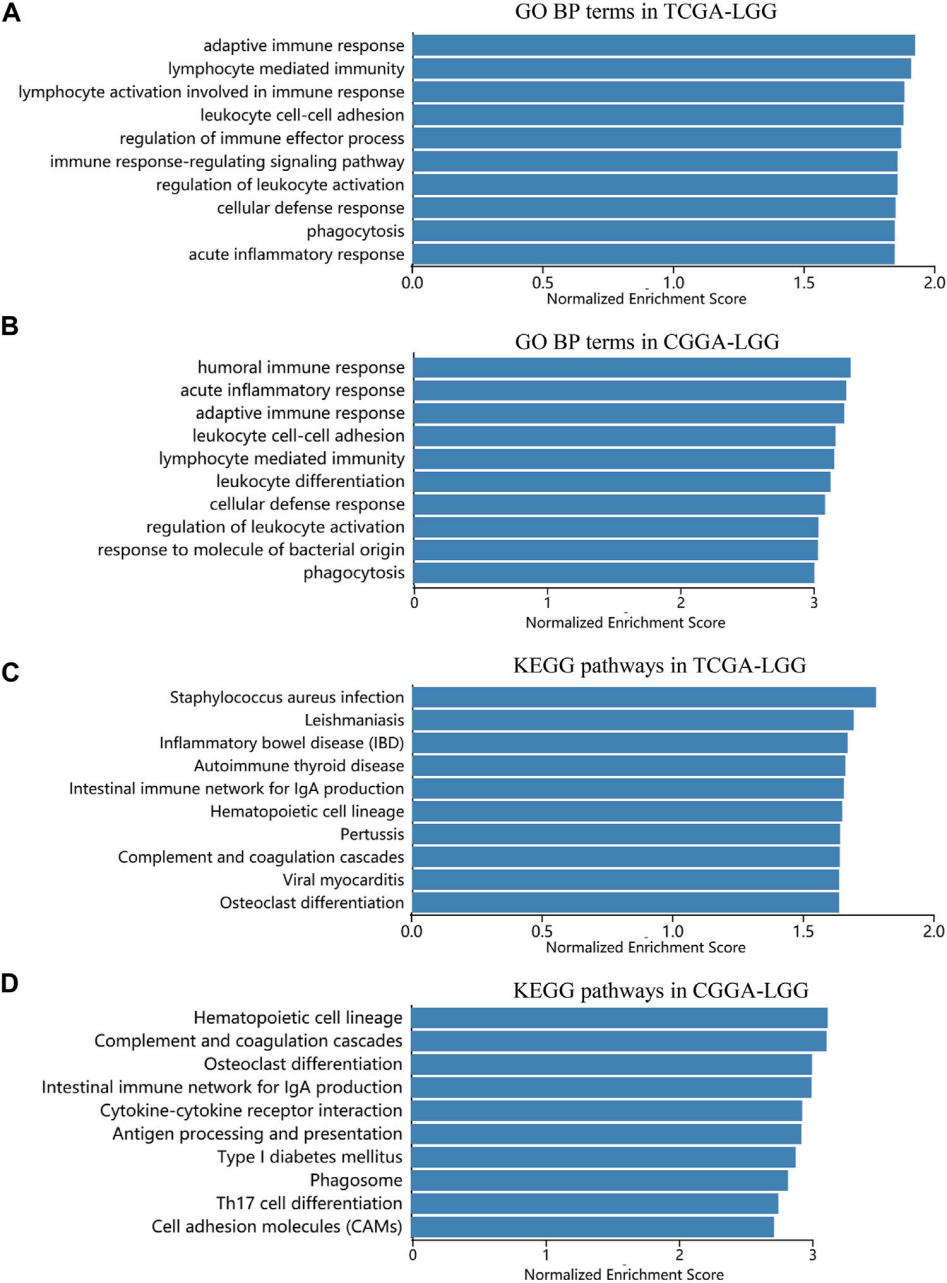
Significant pathways influenced by Siglec-9 based on Gene set enrichment analysis (GSEA) in TCGA-LGG and CGGA-LGG**. (A)** Correlation between Siglec-9 and GO BP terms in LGG from TCGA analyzed by GSEA. **(B)** Correlation between Siglec-9 and GO BP terms in LGG from CGGA analyzed by GSEA. **(C)** Correlation between Siglec-9 and KEGG pathways in LGG from TCGA analyzed by GSEA. **(D)** Correlation between Siglec-9 and KEGG pathways in LGG from CGGA analyzed by GSEA.

To clarify the specific mechanism by which siglec9 affected LGG survival, GSEA analysis was used to identify key biological functions of Siglec-9, between the low and high Siglec-9 subgroups in the TCGA-LGG and CGGA-LGG (WHO grade II and III). Results indicated that various pathways involved in adaptive immune response, cytokine production, inflammatory response, antigen processing, and presentation were enriched in the high Siglec-9 expression subgroup in Gene Ontology (GO) enrichment analysis (Figures 10A,B; Supplementary Figures S10A–F) and KEGG analysis (Figures 10C,D; Supplementary Figures S10G,H). Immune process-related cellular components, such as MHC protein complex, immunological synapse, and secretory granule membrane, were significantly enriched in GO enrichment analysis (Supplementary Figures S10E,F). Interestingly, neural cell function-related pathways were significantly enriched in low Siglec-9 expression in all analyses mentioned above.

## Discussion

In our study, we investigated the expression pattern of Sigelc-9 in TCGA pan-cancer. Abnormal expression of Siglec-9 was observed in the tumor compared with normal tissues in the TCGA and GTEx database. Siglec-9 was upregulated in BRCA, ESCA, GBM, HNSC, KIRC, KIRP, LGG, STAD, UCEC, CESC, LAML, OV, PAAD, TGCT, THCA UCS, SKCM, and STAD (Figure 1), which was similar to the existing result that Siglec-9 was upregulated in colorectal and ovarian cancer (Stanczak et al., 2018). Downregulation of Siglec-9 was observed in ACC, COAD, LIHC, LUAD, LUSC, and PAAD (Figure 1). Differential Siglec-9 expression was observed in different stages and molecular subtypes in various tumors (Figure 2), which indicated that Siglec-9 expression was associated with tumor progression and tumor molecular subtypes. Importantly, high Siglec-9 expression predicted poor prognosis in LGG, LUSC, and THYM, and predicted good prognosis in ACC (Figure 3). The results above indicated that Siglec-9 mRNA expression was correlated with clinical characteristics in multiple tumors and could be a prognostic predictor in ACC, LGG, LUSC, and THYM.

It is well known that Siglec-9 was correlated with the tumor immune microenvironment (Beatson et al., 2016). Siglec-9 has been regarded as a regulator of the immune response in cancers including astrocytoma, epithelial ovarian cancer, colorectal cancer, adenocarcinomas, and hematological cancers (Fraschilla and Pillai, 2017). Our analysis uncovered the correlation between Siglec-9 expression and tumor immune microenvironment or tumor microenvironment in cancers (Figure 4), and further consolidated the pivotal roles of Siglec-9 in tumor immune regulation. Subsequently, we found that Siglec-9 expression was strongly correlated with B cells, CD4^+^ T cells, CD8^+^ T cells, neutrophils, macrophages, and DC cells in most tumors as expected. Other reports have shown that Siglec-9 was expressed on cytotoxic CD8^+^ T cells, neutrophils, and NK cells (Fraschilla and Pillai, 2017). Our findings confirmed the above results to some extent. Notably, Siglec-9 expression was positively correlated with LAIR1, HAVCR2, CD80, PDCD1 (PD1), PDCD1LG2 (PD-L2), VSIR, and CD86 in most tumors (Figure 6E). The upregulation of immune checkpoints and immune suppression molecules, such as LAIR1, HAVCR2, CD80, PD1, and PD-L2, has been described as one of the hallmarks of T-cell exhaustion and suggested T-cell dysfunctional status (Speiser et al., 2016; Thommen and Schumacher, 2018). T-cell exhaustion status is reported to be medication guidance for immune therapy (Thommen and Schumacher, 2018). Based on these findings, we could speculate that Siglec-9 might predict T-cell status according to our result and might be a molecular marker for immune therapy. It was worth pointing out that Siglec-9 had a low correlation with immune checkpoints in ACC, which might result from low expression in ACC and explain the beneficial role of Siglec-9 in the survival analysis of ACC (Figure 3).

The occurrence, progression, and prognosis of tumors were closely related to TMB, MSI, DNMT, and MMRs (Duffy and Crown, 2019). Recent studies have identified TMB, MSI, DNMT, and MMRs as immunotherapy and tumor immune microenvironment-related factors, and may predicate the outcome of immunotherapy (Mandal et al., 2019; Samstein et al., 2019; Segovia et al., 2019). Our study found that Siglec-9 expression was associated with these factors in pan-cancer (Figure 7). Therefore, we speculated that the expression of Siglec-9 could be a predictor of immunotherapy efficacy. There was no direct evidence that the correlations between Siglec-9 expression and these factors would influence the survival of tumor patients. Therefore, the causal relationship still needed to be explored.

Our findings that Siglec-9 expression was significantly correlated with clinical characteristics of LGG suggested the therapeutic roles of Siglec-9 in LGG. IDH mutation status and 1p/19q co-deletion status were beneficial to diagnosis, prognosis prediction, and treatment of glioma (Aoki et al., 2018). The wild type and none 1p/19q co-deletion status was reported to be more insensitive to drugs (Aoki et al., 2018). We found that Siglec-9 expression was higher in wild type and the none 1p/19q co-deletion subtype (Figures 8C,D), suggesting that Siglec-9 expression might participate in drug resistance in LGG. In addition, none 1p/19q co-deletion status was associated with an increased polarization of tumor-associated macrophages toward an M2 phenotype, and most of the TAMs in IDH mutated tumors express M1 activation markers (Zhang et al., 2021). We found that Siglec-9 expression was more positively correlated to M2 than M1 in tumor-associated macrophages just as previously reported (Laubli et al., 2014), suggesting the possible role of Siglec-9 in TAM polarization in LGG. As TAM polarization can exert opposing influence on the effectiveness of cytoreductive therapies (chemotherapy and radiotherapy) (Mantovani et al., 2017), targeting Siglec-9 might benefit cytoreductive therapies for LGG.

GSEA analysis between the low and high Siglec-9 subgroups in the TCGA-LGG and CGGA-LGG showed that various pathways involved in adaptive immune response, cytokine production, inflammatory response, antigen processing, and presentation were enriched in the high Siglec-9 expression subgroup in BP, MF, and KEGG analyses (Figure 10). Immune process-related cellular components, such as MHC protein complex, immunological synapse, and secretory granule membrane, were significantly enriched in CC analysis. Inflammation and adaptive immune response are considered as immunosuppression components in the suppression of effective antitumor immunity during LGG progression (Mantovani et al., 2008; Diakos et al., 2014). Thus, the GSEA results suggested that high Siglec-9 expression may result in activation of adaptive immune response and inflammatory response, and lead to immune suppression status in LGG. Targeting cancer-related inflammation and adaptive immune cells could benefit tumor treatment effectiveness, especially immunotherapy (Diakos et al., 2014). Despite the specific mechanism remaining to be explored and verified, we could speculate that targeting Siglec-9 may benefit immunotherapy of LGG. Interestingly, neural cell function-related pathways were significantly enriched in low Siglec-9 expression, indicating that high Siglec-9 expression was negatively correlated with the normal physiological activity of nerve cells. These results suggest that Siglec-9 may affect the survival time of LGG patients by modulating the immune microenvironment to immunosuppression status.

In conclusion, our study uncovered the correlations between Siglec-9 expression with clinical characteristics of tumor patients including cancer progression, prognosis, and immune status (Supplementary Figure S11). Siglec-9 expression was a prognostic factor for patients with ACC, ESCA, LGG, LUSC, and THYM. A strong correlation was observed between Siglec-9 expression and immune cell infiltration and immune checkpoint expression in most tumors. LGG, ACC, and LUSC showed significance in most analyses, suggesting that Siglec-9 might be more important in LGG, ACC, and LUSC (Supplementary Figure S11). In particular, increased Siglec-9 expression predicted poor prognosis and might influence LGG development through immune regulation and normal physiological activity interference. Collectively, our study indicated that Siglec-9 might be considered as a potential biomarker for predicting prognosis and immune infiltration in multiple tumor patients, especially in LGG. However, all the results above still need further experimental verification.

## Data Availability

The original contributions presented in the study are included in the article/Supplementary Material, further inquiries can be directed to the corresponding authors.
