# Supplementary material for: Siglec-9, a Putative Immune Checkpoint Marker for Cancer Progression Across Multiple Cancer Types
Source: Front Mol Biosci. 2022 Mar 17;9:743515. doi: 10.3389/fmolb.2022.743515 (PMC8968865; doi:10.3389/fmolb.2022.743515)
Supplement: Supplementary file 1 [file DataSheet1.PDF]

Supplementary Material

1 Supplementary Figures and Tables

1.1 Supplementary Figures

Supplementary Figure 1

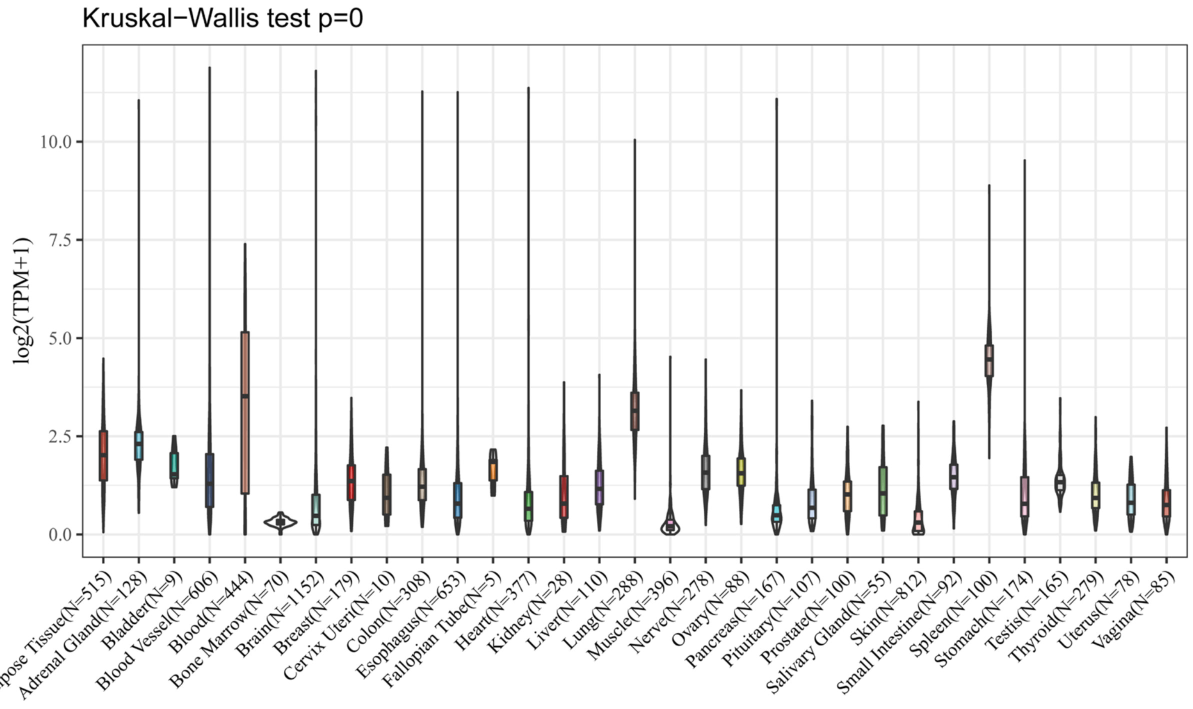

Supplementary Figure 1. mRNA expression of Siglec-9 in normal tissue obtained from GTEx database.

Supplementary Figure 2

A

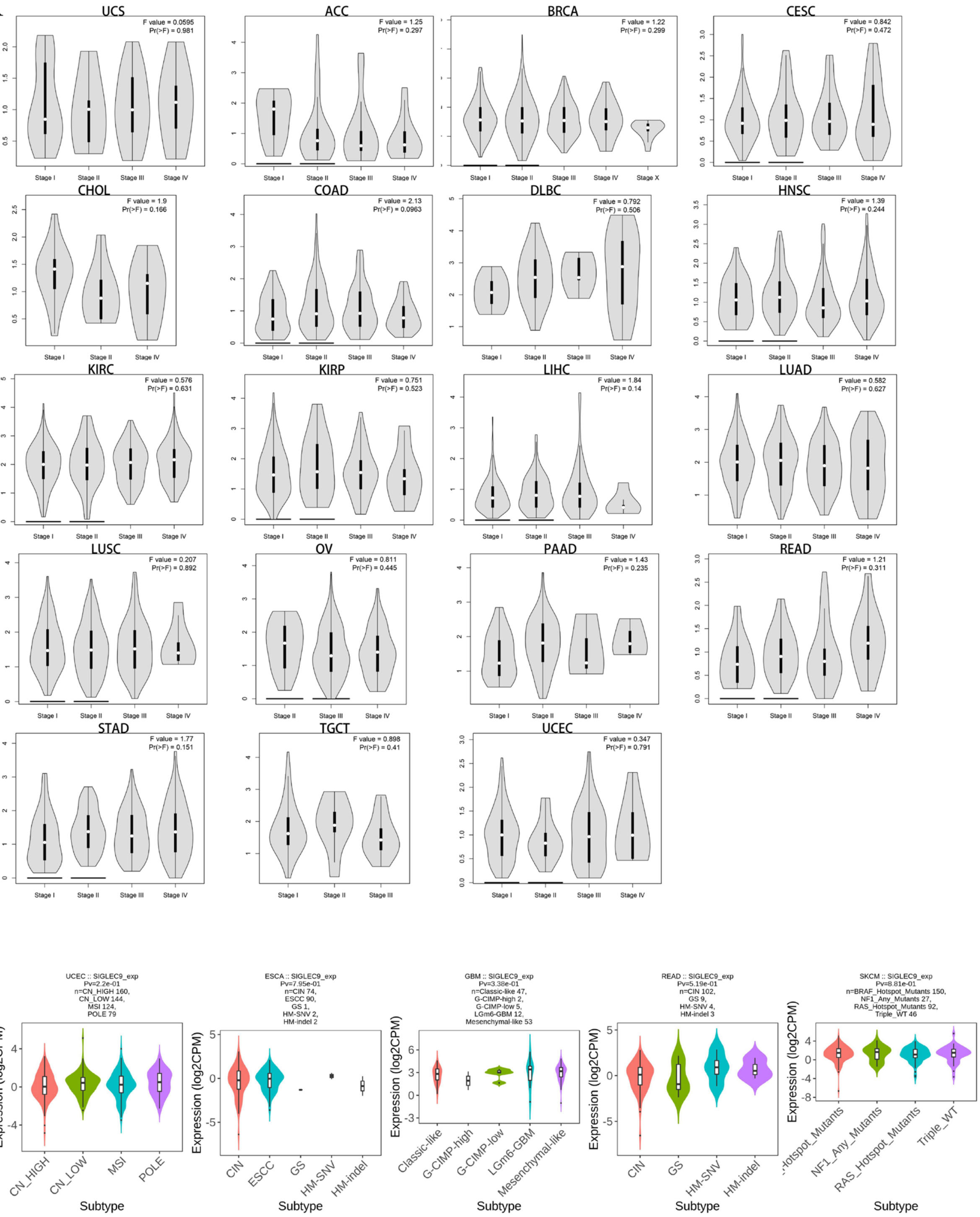

Supplementary Figure 2. The relationship between Siglec-9 expression and tumor stages and molecular subtypes based on GEPIA2.0 and TISIDB. ($p<0.05$ was considered significant)

Supplementary Figure 3

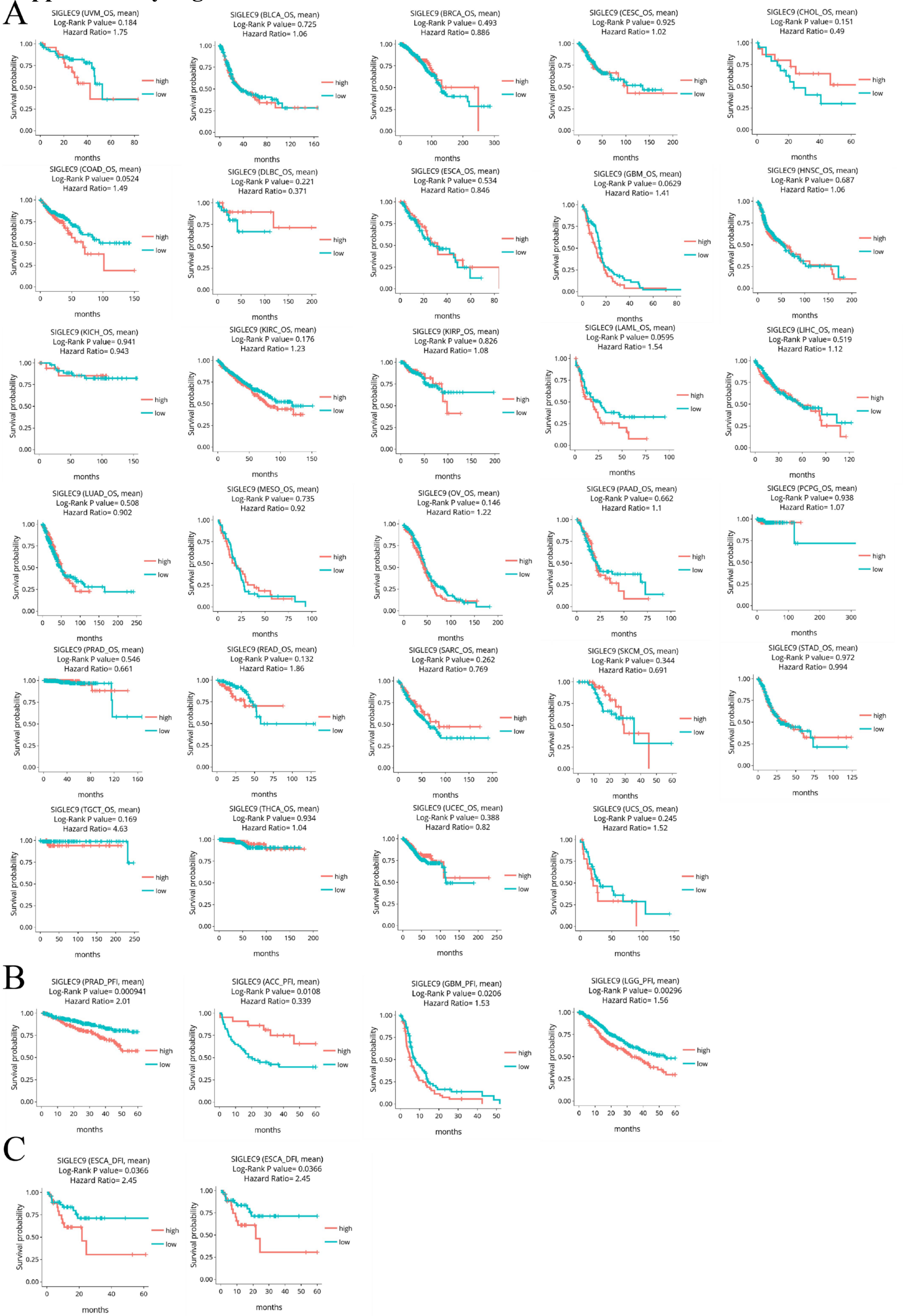

Supplementary Figure 3. Correlation between Siglec-9 expression and prognostic value relied on DriverDBv3. **(A)** Association between Siglec-9 expression and OS in the rest tumors. **(B)** Siglec-9 was associated with 5-years PFI. **(C)** Siglec-9 was associated with DFI and 5-years DFI in ESCA. (p<0.05 was considered significant)

Supplementary Figure 4

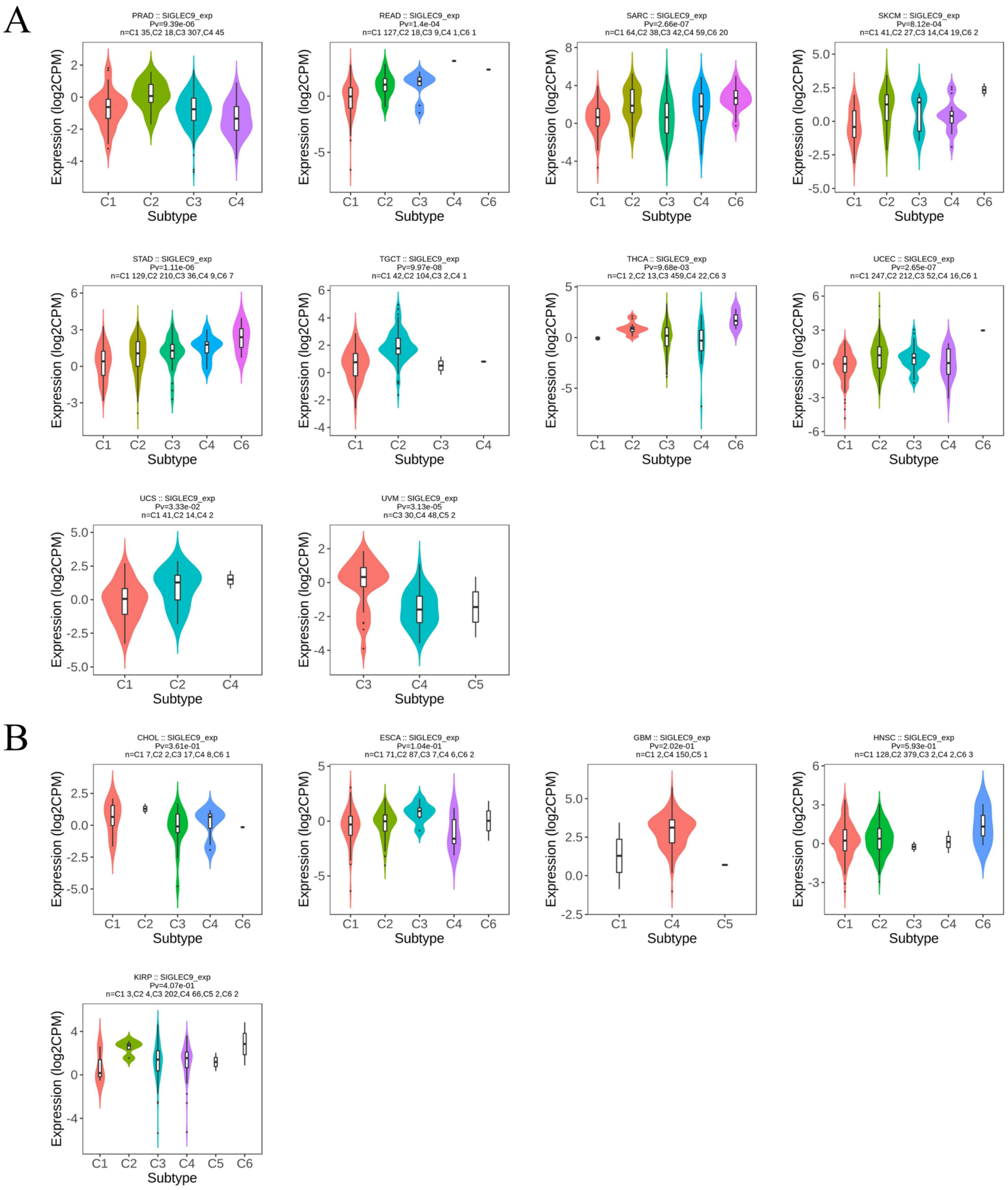

Supplementary Figure 4. The significant correlation (A) and nonsignificant (B) between Siglec-9 mRNA expression and immune subtypes across tumor types.

Supplementary Figure 5

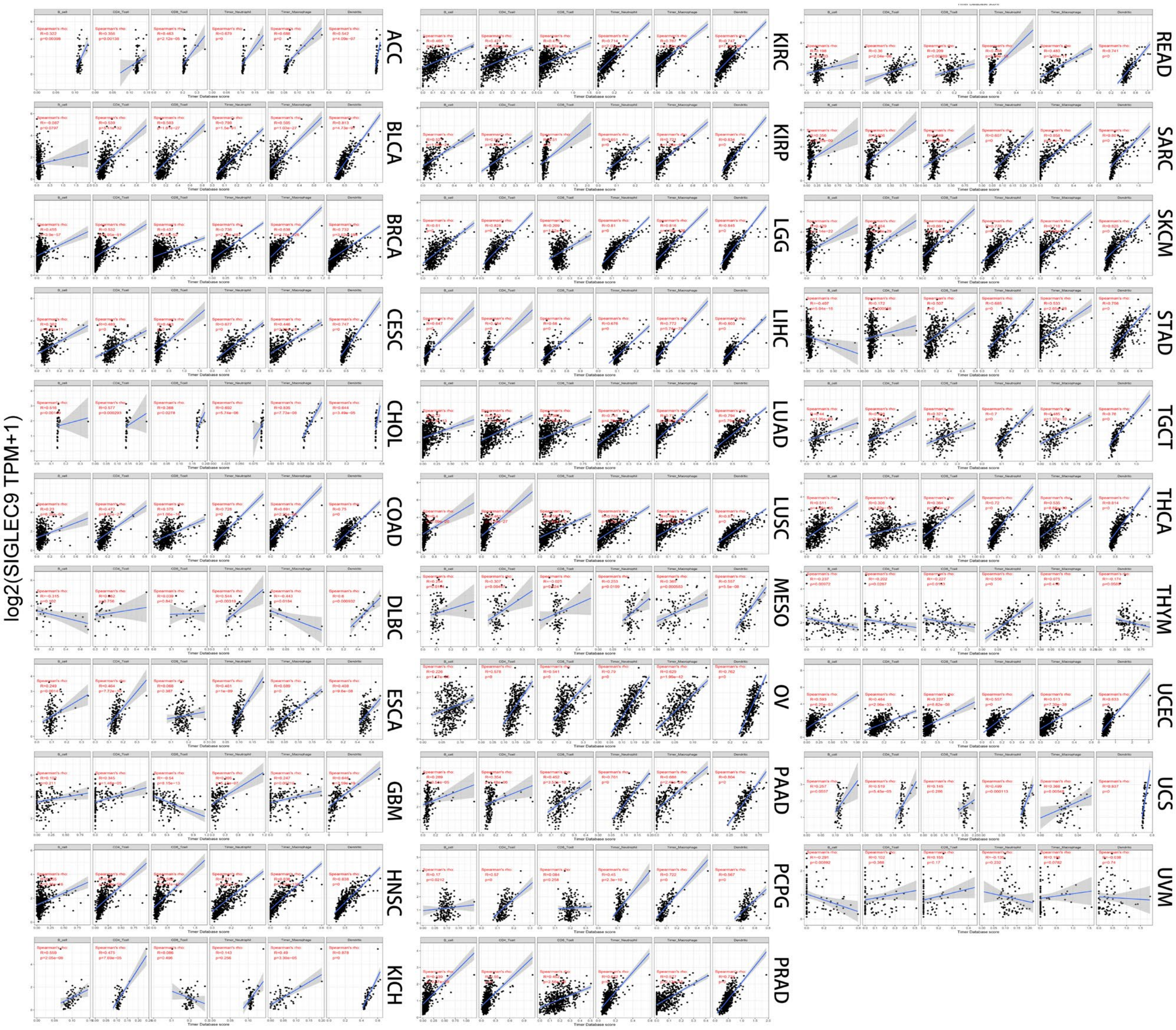

Supplementary Figure 5. Relationship between Siglec-9 expression and immune cell infiltration. Relationship between immune cell infiltration (B cell, CD4+ T cell, CD8+ T cell, Neutrophil, Macrophage, Dendritic cell) and Siglec-9 expression in various cancer. (Spearman Correlation test, p<0.05 was considered to be significant.)

Supplementary Figure 6

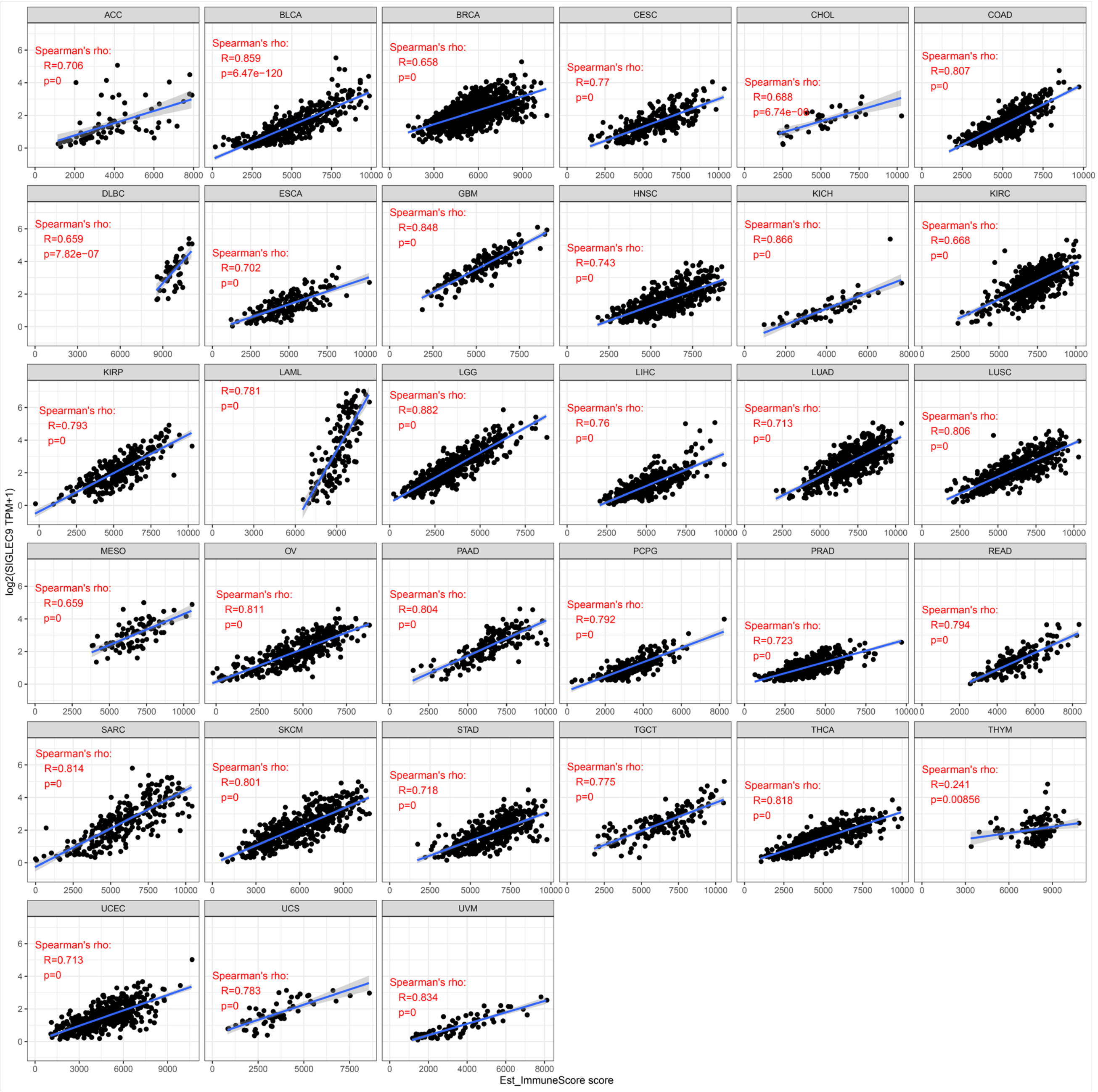

Supplementary Figure 6. Relationships between Siglec-9 expression and Immune Score in different cancer. (Spearman Correlation test, p<0.05 was considered to be significant.)

Supplementary Figure 7

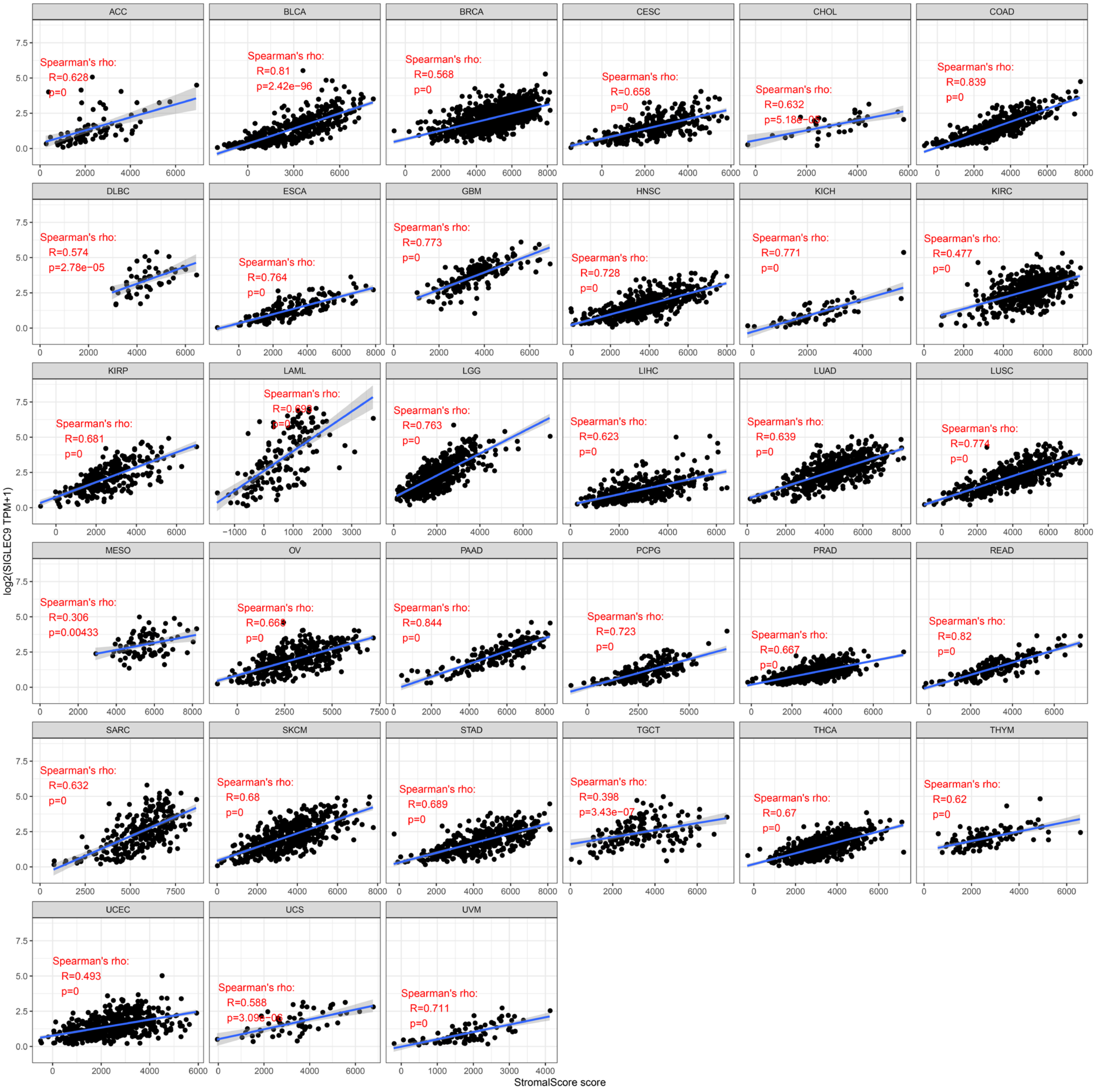

Supplementary Figure 7. Relationships between Siglec-9 expression and Stromal Score in different cancer. (Spearman Correlation test, p<0.05 was considered to be significant.)

Supplementary Figure 8

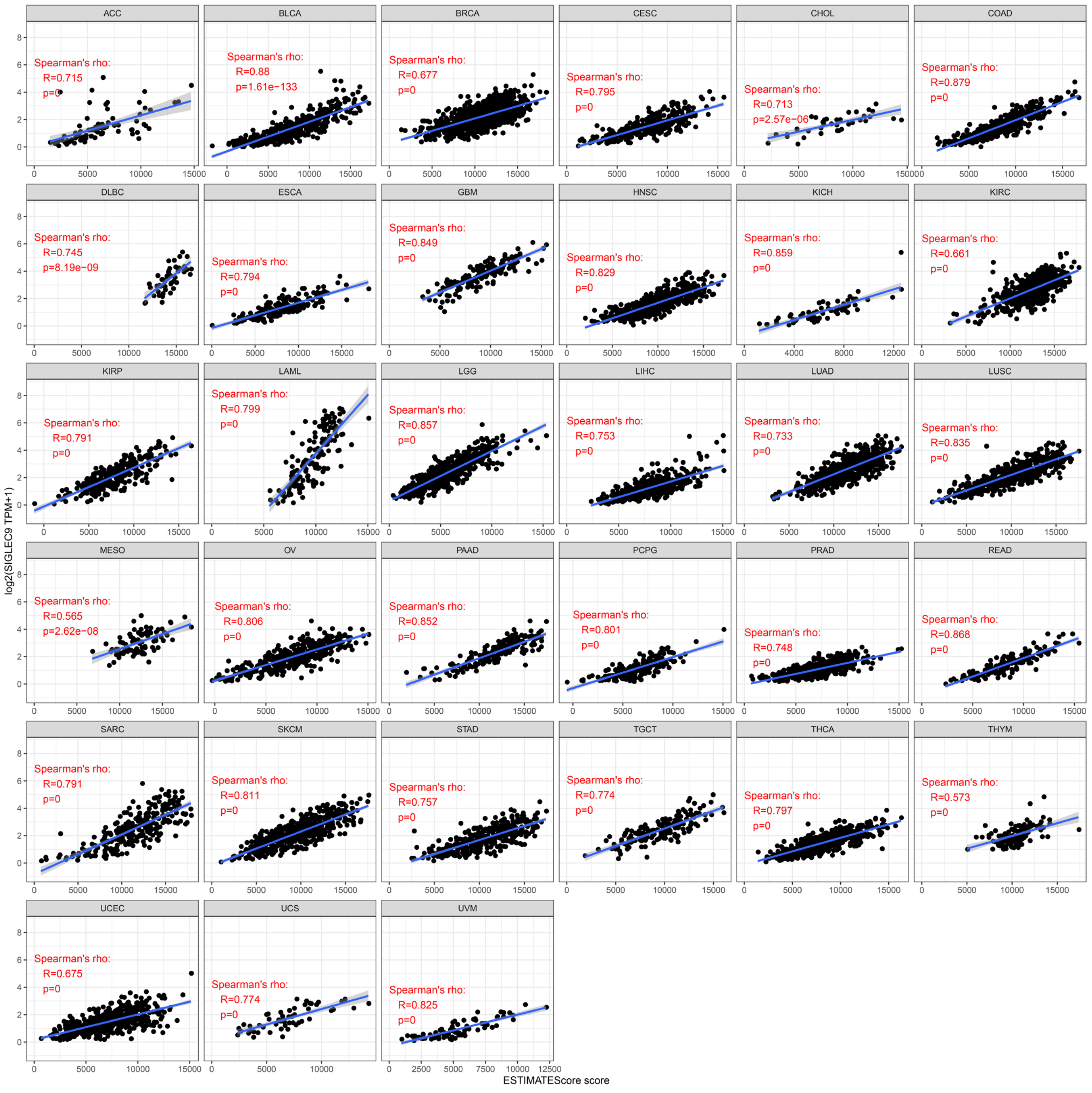

Supplementary Figure 8. Relationships between Siglec-9 expression and ESTIMATE Score in different cancer. (Spearman Correlation test, p<0.05 was considered to be significant.)

Supplementary Figure 9

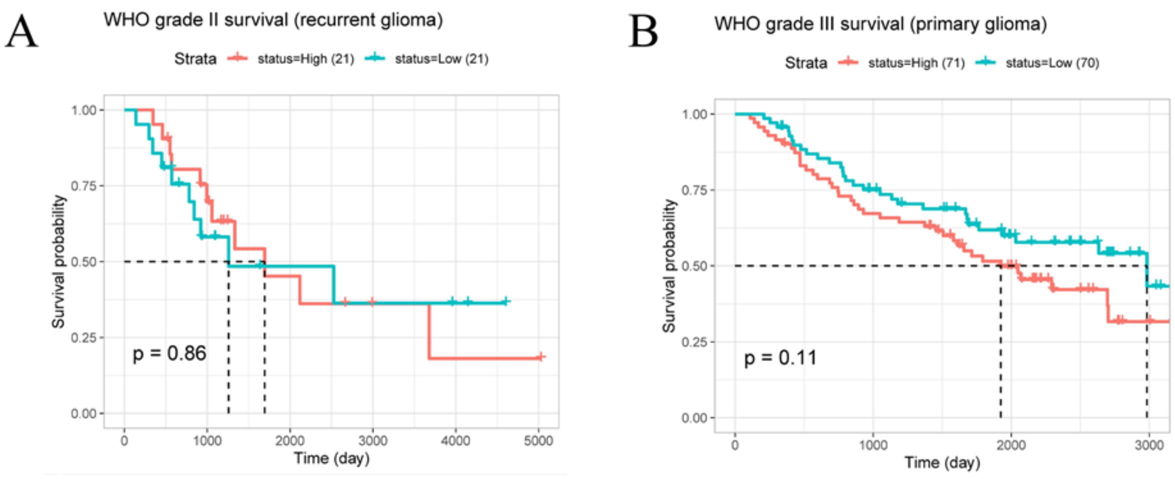

Supplementary Figure 9. K-M survival analysis in WHO II recurrent glioma (A) and WHO III primary glioma (B).

Supplementary Figure 10

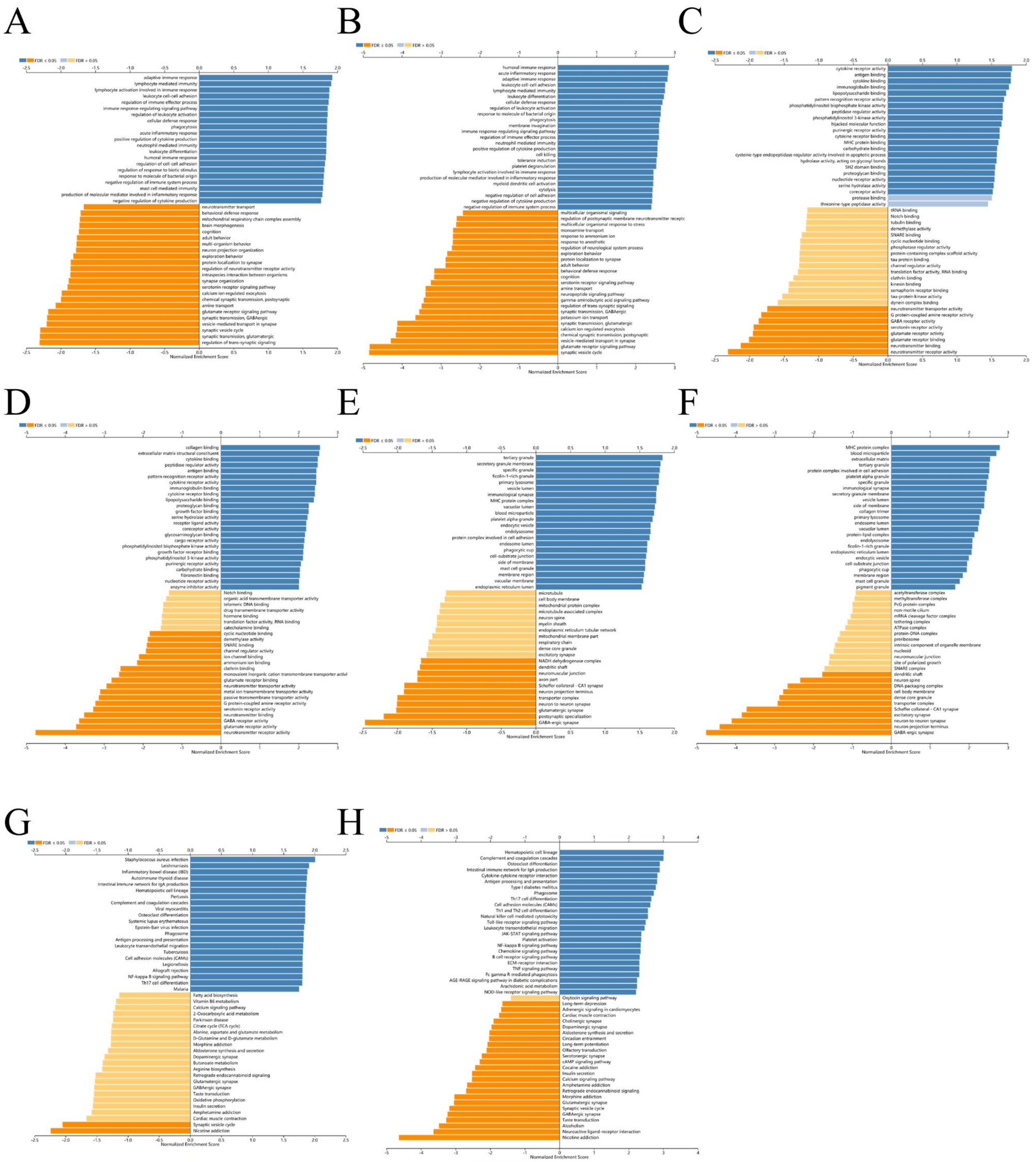

Supplementary Figure 10. Significant pathways influenced by Siglec-9 based on Gene set enrichment analysis (GSEA) in TCGA-LGG and CGGA-LGG**. (A)** Correlation between Siglec-9 and GO BP terms in LGG from TCGA analyzed by GSEA. **(B)** Correlation between Siglec-9 and GO BP terms in LGG from CGGA analyzed by GSEA. **(C)** Correlation between Siglec-9 and GO MF terms in LGG from TCGA analyzed by GSEA. **(D)** Correlation between Siglec-9 and GO MF terms in LGG from CGGA analyzed by GSEA. **(E)** Correlation between Siglec-9 and GO CC terms in LGG

from TCGA analyzed by GSEA. **(F)** Correlation between Siglec-9 and GO CC terms in LGG from CGGA analyzed by GSEA. **(G)** Correlation between Siglec-9 and KEGG pathways in LGG from TCGA analyzed by GSEA. **(H)** Correlation between Siglec-9 and KEGG pathways in LGG from CGGA analyzed by GSEA.

Supplementary Figure 11

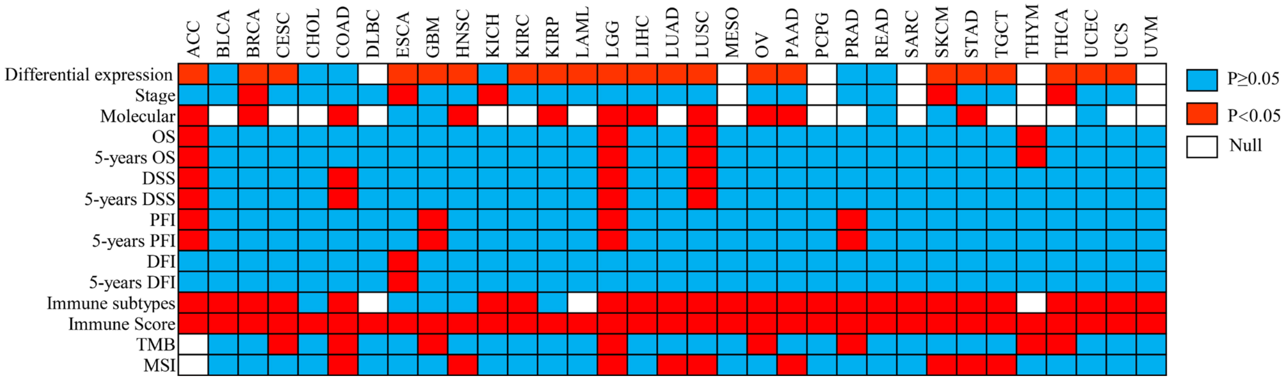

Supplementary Figure 11. The overall role of Siglec-9 in pan-cancer visualized with the main result of pan-cancer analyses in this article. (OS: overall survival; DSS: disease-specific survival; DFI: disease-free interval; TMB: Tumor Mutation Burden; MSI: Microsatellite Instability; Null: data missed.)

1.2 Supplementary Tables

Supplementary Table 1

Supplementary Table1. Disease-specific survival analysis of TCGA tumors by DriverDBv3.

Cancer type	Overall survival		5-years survival		Num. of high expressed example	Num. of low expressed example
	Log-rank p-value	HR	Log-rank p-value	HR		
GBM	0.0941	1.39	0.114	1.37	61	79
OV	0.276	1.18	0.377	1.15	137	210
LUAD	0.387	0.842	0.232	0.783	183	282
LUSC	0.0374 (*)	1.57	0.0175 (*)	1.71	158	284
PRAD	0.369	0.38	0.158	2.38E-09	190	303
UCEC	0.479	0.819	0.47	0.811	184	355
BLCA	0.453	0.859	0.508	0.873	115	277
TGCT	0.169	4.63	0.169	4.63	40	94
ESCA	0.716	1.12	0.716	1.12	58	102
PAAD	0.44	1.2	0.44	1.2	70	100
KIRP	0.494	0.73	0.55	0.759	83	198
CESC	0.383	0.771	0.558	0.838	101	186
LIHC	0.869	0.962	0.802	0.94	122	235
SARC	0.481	0.837	0.618	0.875	79	174
BRCA	0.492	0.85	0.995	1	416	641
THYM	0.202	3.93	0.0963	8.65E+08	47	71
MESO	0.464	0.795	0.464	0.795	26	38
COAD	0.0339 (*)	1.73	0.0321 (*)	1.76	148	274
STAD	0.351	0.812	0.343	0.807	126	206
CHOL	0.106	0.425	0.159	0.466	15	20
KIRC	0.0593	1.44	0.0684	1.44	214	303
THCA	0.654	0.689	0.654	0.689	179	316
HNSC	0.859	0.968	0.799	0.953	166	308
READ	0.258	1.85	0.258	1.85	57	96
SKCM	0.311	0.627	0.311	0.627	38	64
LGG	0.000215 (***)	1.99	0.000606 (***)	2.04	172	326
DLBC	0.903	0.884	0.903	0.884	21	26
KICH	0.746	1.31	0.564	1.64	18	46
UCS	0.425	1.35	0.484	1.32	19	34
ACC	0.00908 (**)	0.181	0.0247 (*)	0.22	21	56
PCPG	0.8	0.745	0.969	0.954	62	116
UVM	0.202	1.76	0.202	1.76	28	52

*: P<0.05; **:P<0.01; ***:P<0.001

Supplementary Table 2

Supplementary Table2. Progression-free interval analysis of TCGA tumors by DriverDBv3.

Cancer type	Overall survival		5-years survival		Num. of high expressed example	Num. of low expressed example
	Log-rank p-value	HR	Log-rank p-value	HR		
GBM	0.0206 (*)	1.53	0.0206 (*)	1.53	68	85
OV	0.846	1.02	0.916	1.01	144	228
LUAD	0.322	0.866	0.27	0.85	200	300
LUSC	0.241	1.23	0.163	1.28	174	321
PRAD	0.0016 (**)	1.91	0.000941 (***)	2.01	192	303
UCEC	0.331	0.828	0.232	0.787	185	356
BLCA	0.59	0.914	0.728	0.943	119	288
TGCT	0.633	0.831	0.646	0.827	40	94
ESCA	0.608	1.13	0.608	1.13	58	103
PAAD	0.727	1.07	0.727	1.07	72	104
KIRP	0.504	0.813	0.791	0.92	85	198
CESC	0.393	0.803	0.433	0.817	102	189
LIHC	0.287	0.843	0.262	0.835	124	242
SARC	0.809	0.956	0.978	1.01	80	179
BRCA	0.777	1.05	0.318	1.2	422	654
THYM	0.44	1.41	0.657	1.24	47	71
MESO	0.852	0.951	0.958	0.986	31	51
COAD	0.104	1.36	0.144	1.33	155	282
STAD	0.174	0.772	0.195	0.78	136	219
CHOL	0.286	0.615	0.286	0.615	15	21
KIRC	0.548	1.1	0.655	1.08	218	308
THCA	0.472	1.22	0.472	1.22	179	322
HNSC	0.388	0.876	0.273	0.842	177	322
READ	0.976	1.01	0.976	1.01	58	100
SKCM	0.881	0.95	0.881	0.95	38	64
LGG	0.00243 (**)	1.56	0.00296 (**)	1.56	176	330
DLBC	0.752	0.814	0.801	1.19	21	26
KICH	0.848	1.14	0.412	1.81	18	46
UCS	0.718	1.14	0.862	1.07	19	36
ACC	0.0157 (*)	0.379	0.0108 (*)	0.339	22	57
PCPG	0.727	0.842	0.621	0.769	62	115
UVM	0.748	1.13	0.602	1.22	30	49

*: P<0.05; **:P<0.01; ***:P<0.001

Supplementary Table 3

Supplementary Table3. Disease-free interval analysis of TCGA tumors by DriverDBv3.

Cancer type	Overall survival		5-years survival		Num.of high expressed example	Num. of low expressed example
	Log-rank p-value	HR	Log-rank p-value	HR		
OV	0.859	1.03	0.962	0.991	68	109
LUAD	0.544	0.873	0.514	0.862	119	180
LUSC	0.998	0.999	0.794	1.08	107	193
PRAD	0.293	1.47	0.293	1.47	130	207
BLCA	0.37	0.701	0.5	0.765	61	125
TGCT	0.883	1.06	0.811	1.12	31	74
PAAD	0.486	1.36	0.486	1.36	30	39
KIRP	0.652	1.21	0.291	1.6	54	128
CESC	0.741	0.869	0.844	0.919	60	114
LIHC	0.232	0.804	0.221	0.799	102	211
SARC	0.965	1.01	0.652	1.13	48	104
BRCA	0.526	1.15	0.441	1.2	370	566
COAD	0.644	0.803	0.706	0.835	59	128
STAD	0.776	1.1	0.776	1.1	86	127
CHOL	0.849	0.884	0.849	0.884	9	15
KIRC	0.634	0.771	0.644	0.754	49	66
UCEC	0.53	0.834	0.429	0.792	139	283
READ	0.914	0.915	0.914	0.915	22	25
HNSC	0.158	1.71	0.202	1.64	42	82
THCA	0.812	1.1	0.812	1.1	125	227
LGG	0.436	0.67	0.436	0.67	41	90
DLBC	0.558	0.408	0.317	1.62E+09	14	14
ESCA	0.0366 (*)	2.45	0.0366 (*)	2.45	26	47
KICH	0.98	1.03	0.157	4.17E+09	10	18
MESO	0.423	1.91	0.423	1.91	5	8
UCS	0.924	0.926	0.565	0.542	7	18
ACC	0.513	0.644	0.284	0.438	15	30

*: $P<0.05$

Supplementary Table 4

Supplementary Table4. Overall survival analysis of TCGA tumors by UALCAN.

Cancer type	Log-rank p-value	Prognosis (poor/good)	Num.of high expressed example	Num. of low expressed example
ACC	0.057	-	20	59
BLCA	0.82	-	101	305
BRCA	0.72	-	267	814
CESC	0.44	-	73	218
CHOL	0.21	-	9	27
COAD	0.13	-	71	208
DLBC	0.75	-	12	35
ESCA	0.87	-	46	138
GBM	0.4	-	39	113
HNSC	0.37	-	130	389
KICH	0.97	-	17	48
KIRC	0.86	-	134	397
KIRP	0.99	-	71	216
LAML	0.59	-	44	119
LGG	0.0001 (***)	poor	128	383
LIHC	0.069	-	90	275
LUAD	0.31	-	124	378
LUSC	0.09	-	125	369
MESO	0.66	-	21	64
OV	0.37	-	76	227
PAAD	0.6	-	45	132
PCPG	0.55	-	45	134
PRAD	0.85	-	125	372
READ	0.02 (*)	good	42	123
SARC	0.27	-	65	194
SKCM	0.0018 (**)	poor	117	342
TGCT	0.12	-	35	99
THCA	0.25	-	127	377
THYM	0.18	-	29	90
UCEC	0.4	-	137	406
UCS	0.21	-	14	42
UVM	0.036 (**)	poor	20	60

*: P<0.05; **:P<0.01; ***:P<0.001
